# ABA-Dependent and ABA-Independent Functions of RCAR5/PYL11 in Response to Cold Stress

**DOI:** 10.3389/fpls.2020.587620

**Published:** 2020-09-25

**Authors:** Chae Woo Lim, Sung Chul Lee

**Affiliations:** Department of Life Science (BK21 Program), Chung-Ang University, Seoul, South Korea

**Keywords:** abscisic acid, cold stress, RCAR, seed germination, stomatal closure

## Abstract

*Arabidopsis thaliana* has 14 abscisic acid (ABA) receptors—PYR1/PYLs/RCARs—which have diverse and redundant functions in ABA signaling; however, the precise role of these ABA receptors remains to be elucidated. Here, we report the functional characterization of RCAR5/PYL11 in response to cold stress. Expression of *RCAR5* gene in dry seeds and leaves was ABA-dependent and ABA-independent, respectively. Under cold stress conditions, seed germination was negatively affected by the level of *RCAR5* expression, which was dependent on ABA and was regulated by HAB1, OST1, and ABI5—downstream components of RCAR5 in ABA signaling. Leaves of *RCAR5*-overexpressing plants showed enhanced stomatal closure—independent of ABA—and high expression levels of cold, dehydration, and/or ABA-responsive genes compared to those of wild-type; these traits conferred enhanced freezing tolerance. Our data suggest that RCAR5 functions in response to cold stress by delaying seed germination and inducing rapid stomatal closure *via* ABA-dependent and ABA-independent pathways, respectively.

## Introduction

The plant hormone abscisic acid (ABA) plays a key role in growth and development, as well as in adaptive mechanisms to unfavorable conditions such as regulation of seed dormancy, germination, and stomatal opening and closure (Cutler et al., 2010; Hubbard et al., 2010). During seed maturation, ABA accumulates in seeds, inducing and maintaining seed dormancy and inhibiting seed germination by preventing water uptake and endosperm rupture (Müller et al., 2006; Seo et al., 2006). Seed germination leads to a rapid decline in ABA content and suppression of ABA signaling, suggesting that ABA is an important hormone inhibiting seed germination until favorable growth conditions prevail (Finkelstein et al., 2008; Shu et al., 2016). In vegetative tissue, drought stress induces ABA accumulation, leading to stomatal closure and induction of stress-related genes (Cutler et al., 2010). In *Arabidopsis*, ABA levels increase transiently and less markedly (2-fold) in response to cold stress than in response to drought stress (approximately 20-fold) (Lång et al., 1994). Several studies have shown that ABA is involved in cold stress responses; these studies have examined cold stress-induced ABA biosynthesis (Lång et al., 1994; Cuevas et al., 2008); absence of cold acclimation in ABA-deficient mutants (Gilmour and Thomashow, 1991; Xiong et al., 2001); and ABA induction of cold-responsive genes, mainly CBF genes (Knight et al., 2004; Lee and Seo, 2015). However, in comparison with the well documented role of ABA in drought stress, the function of this plant hormone in cold stress and acclimation remains controversial.

ABA is perceived by the PYRABACTIN RESISTANCE/PYRABACTIN RESISTANCE-LIKE/REGULATORY COMPONENT OF ABA RECEPTOR (PYR/PYL/RCAR; hereafter referred to as RCARs) protein family, which consists of 14 members in *Arabidopsis*. RCARs belong to the START-domain superfamily and are divided into three subfamily groups according to sequence homology (Ma et al., 2009; Park et al., 2009). In response to environmental stresses, ABA is rapidly synthesized and binds to RCARs (Cutler et al., 2010). ABA-bound RCARs selectively interact with and inhibit clade A protein phosphatase 2Cs (PP2Cs), including ABA-insensitive 1 (ABI1), ABI2, hypersensitive to ABA1 (HAB1), HAB2, ABA-hypersensitive germination 1 (AHG1), AHG3/PP2CA, highly ABA-induced PP2C gene 1 (HAI1), HAI2, and HAI3 (Ma et al., 2009; Park et al., 2009; Nishimura et al., 2010; Bhaskara et al., 2012; Gonzalez-Guzman et al., 2012). Among potential RCAR–PP2C interactions, 113 pairings are known to be functional (Tischer et al., 2017). As a consequence of these interactions, PP2C inhibition of sucrose non-fermenting 1-related protein kinase 2s (SnRK2s) is canceled, resulting in phosphorylation and activation of downstream components, such as transcription factors and ion channels (Furihata et al., 2006; Geiger et al., 2009; Lee et al., 2009). In this process, bZIP transcription factor ABI5—the core ABA signaling component—is phosphorylated by ABA-activated SnRK2.2, SnRK2.3, and SnRK2.6 (Nakashima et al., 2009) and subsequently regulates expression of stress adaptation genes, e.g., late embryonic and abundant genes such as *EARLY METHIONINE-LABELED 1* (*EM1*) and *EM6* (Finkelstein and Lynch, 2000).

RCARs have diverse and redundant functions in ABA and drought-stress signaling (Hao et al., 2011; Antoni et al., 2013; Zhao et al., 2013; Zhao et al., 2016). With the exception of RCAR7, RCARs induce expression of ABA-responsive genes with highly variable expression levels. Also, RCAR levels—with the exception of RCAR4—significantly suppressed by ABA deficiency (Fujii et al., 2009; Ma et al., 2009; Szostkiewicz et al., 2010; Zhao et al., 2013; Tischer et al., 2017). This may be explained by differing ABA affinities and ABA dependencies of RCARs and/or variations in abundance of RCARs and their target PP2Cs (Tischer et al., 2017). ABA affinities of RCARs are influenced by oligomerization states; monomeric RCARs (RCAR1, RCAR3, RCAR4, RCAR8, RCAR9, and RCAR10) have higher affinities than dimeric RCARs (RCAR11, RCAR12, RACR13, and RCAR14) (Dupeux et al., 2011; Hao et al., 2011). Moreover, monomeric RCARs interact with PP2Cs mainly in an ABA-independent manner, whereas dimeric RCARs bind to PP2Cs in an ABA-dependent manner (Hao et al., 2011; Tischer et al., 2017). In most RCARs, functional redundancy interferes with analysis of biological function in ABA signaling based on single-gene mutations (Park et al., 2009). In contrast, mutation in multiple *RCARs* negatively influences ABA sensitivity and drought resistance, whereas overexpression of *RCARs* positively regulates ABA signaling and drought response (Santiago et al., 2009; Gonzalez-Guzman et al., 2012; Lim et al., 2014; Zhao et al., 2014). Loss-of-function of *RCAR3/PYL8* resulted in reduced sensitivity to ABA-induced inhibition of primary root growth (Antoni et al., 2013). In combination with *RCAR2/PYL9* and *RCAR3/PYL8* functions in regulating of lateral root growth *via* interaction with MYB transcription factor MYB77 and MYB44 by independent manner of the core ABA signaling pathway (Zhao et al., 2014; Xing et al., 2016). *RCAR2/PYL9* overexpression conferred drought resistance by promoting ABA-mediated leaf senescence (Zhao et al., 2016). This suggests that RCARs have distinct functions, which are associated with different tissues, developmental stages, and specific environmental conditions (Sun et al., 2017). However, non-redundant functionality of RCAR in an ABA-dependent and/or ABA-independent manner remains to be elucidated. Here, we attempted to functionally characterize RCAR5 in response to cold stress.

## Materials and Methods

### Plant Material and Growth Conditions

Here, *A. thaliana* (ecotype Col-0 and Ler) plants were used as the wild-type (WT). The T-DNA insertion mutants *rcar1* (SALK_083621; Antoni et al., 2013), *rcar2* (SALK_012096; Antoni et al., 2013), *rcar3* (SALK_033867; Antoni et al., 2013), *abi5-8* (SALK_013163; Zheng et al., 2012), *hab1* (SALK_002104; Saez et al., 2004), and *pp2ca-1* (SALK_124564; Lim et al., 2014), *ost1-3* (srk2e, SALK_008068; Yoshida et al., 2002), and the EMS-mutagenized mutant *aba1-3* (Ler background; Koornneef et al., 1982) and *aba1-6* (Col-0 background; Niyogi et al., 1998), were obtained from the Arabidopsis Biological Resource Center (ABRC). Plants were routinely grown in soil mixture containing a 9:1:1 ratio of peat moss, perlite and vermiculite. The plants were maintained at 24°C and 60% humidity under fluorescent light (130 μmol photons·m^-2^·s^-1^) with a 16 h light/8 h dark cycle. Prior to *in vitro* culture, seeds of *Arabidopsis* were surface sterilized with 70% ethanol for 1 min and treated with 2% sodium hydroxide for 10 min. The seeds were then washed 10 times with sterile distilled water and sown on MS agar plates (Sigma, St. Louis, MO) supplemented with 1% sucrose. Following stratification at 4°C for 2 days in the dark, the plates were sealed and incubated at 24°C in a chamber under fluorescent light (130 μmol photons·m^-2^·s^-1^) with a 16 h light/8 h dark cycle. For tobacco (*Nicotiana benthamiana*) plants, seeds were sown in a steam-sterilized compost soil mix (peat moss, perlite, and vermiculite, 5:3:2, v/v/v), sand, and loam soil (1:1:1, v/v/v). The tobacco plants were raised in a growth chamber at 25 ± 1°C under the conditions described above.

### Generation of Transgenic *RCAR*-Overexpressing Mutants and *RCAR5* RNAi Mutant

We previously generated *Arabidopsis* transgenic lines overexpressing *RCAR2* (Lee et al., 2013), *RCAR3* (Lim et al., 2014), *RCAR4*, and *RCAR5* (Lim and Lee, 2015). Here, we generated transgenic lines overexpressing each of the remaining *RCAR* gene family members in *Arabidopsis*. The coding sequences of the 10 *RCARs* were cloned into the pENTR/D-TOPO vector (Invitrogen, Carlsbad, CA, USA) and integrated into pK2GW7 using the LR reaction to induce constitutive expression of each *RCAR* under the control of the cauliflower mosaic virus 35S promoter (Karimi et al., 2002). For *RCAR5* RNAi construct, 221-base pair (from 1 to 221 nt) DNA fragment was amplified. Gateway cassette for RNAi was made using pANIC12A (obtained from ABRC) and integrated into pK2GW7. The correct construct was introduced into *Agrobacterium tumefaciens* strain GV3101 *via* electroporation. We conducted *Agrobacterium*-mediated transformation using the floral dip method (Clough and Bent, 1998). For selection of transgenic lines, seeds harvested from the putative transformed plants were plated on MS agar plates containing 50 μg ml^–1^ of kanamycin or 25 μg ml^–1^ of phosphinothricin.

### Cold Treatment and Phenotypic Analyses

To measure the germination rate of WT and transgenic plants under cold stress conditions, seeds were vernalized at 4°C for 2 days and continuously incubated in darkness until the indicated time points. To analyze whether cold sensitivity of *Arabidopsis* plants is associated with ABA signaling, seeds were plated on MS agar medium supplemented with ABA (0.75 μM) and/or norflurazon (NF; 25 or 50 μM) as the ABA biosynthesis inhibitor.

For thermal imaging analysis, 2-week-old *Arabidopsis* seedlings were subjected to cold stress by exposure to 4 ± 1°C until the indicated time points. Thermal images were obtained using an infrared camera (FLIR systems; T420), and leaf temperatures were measured with FLIR Tools+ version 5.2 software.

For measuring the water loss rates at low temperatures, aerial parts were collected from 3-week-old *Arabidopsis* seedlings of each plant lines (n = 30) at 2, 4, 6, and 24 h after exposure to 4 ± 1°C, and its fresh weights were measured. Water loss rates of the treated samples were calculated by setting the fresh weights of non-treated WT plants to 100%.

For freezing tolerance assays, 3-week-old *Arabidopsis* seedlings were exposed to 4 ± 1°C for 0.5 h, followed by 1°C for 1 h. To set experimental temperatures, the temperature was incrementally decreased by 2°C over a period of 30 min and maintained for 1 h. After freezing treatment, plants were incubated at 4 ± 1°C in the dark for 12 h and then transferred to normal growth conditions. The survival rate after 4 days was measured.

### Measurement of ABA Content

ABA content was measured as described previously (Lim and Lee, 2016). The ABA content of the rosette leaves from *Arabidopsis* treated with dehydration and cold stress was quantified using the Phytodetek-ABA kit (Agdia Inc., Elkhart, IN, USA), according to the manufacturer’s instructions.

### Electrolyte Leakage Test

Electrolyte leakage of WT and *Pro35S:RCAR5* transgenic plants subjected to freezing stress was measured as described previously, but with modifications to sample type and incubation time (Ding et al., 2015). For cold acclimation, 2-week-old plants grown in soil were treated at 4 ± 1°C for 2 days and then exposed to −4°C for 1 h prior to an electrolyte leakage test. Leaf samples were detached from each plant line and placed into tubes containing 20 mL of deionized water (S0). Following shaking for 12 h at room temperature, the conductivity of the samples was measured (S1). After autoclaving for 15 min, the tubes were shaken for 1 h and the conductivity was measured (S2). Electrolyte leakage of each sample was calculated using the formula S1 – S0/S2 – S0.

### RNA Isolation and Quantitative RT-PCR

Total RNA isolation and quantitative RT-PCR analysis were performed as described previously (Lim and Lee, 2016). Especially, total RNA was extracted from *Arabidopsis* seeds using TaKaRa MiniBEST plant RNA extraction kit (TaKaRa Bio Inc., Japan) according to the manufacturer’s instructions. Two-week-old WT and transgenic plants were subjected to cold stress (4°C), and leaf samples were harvested at the indicated time points. cDNA was synthesized using a Transcript First Strand cDNA Synthesis kit (Roche, Indianapolis, IN) with 1 µg of total RNA according to the manufacturer’s instructions. For quantitative RT-PCR (qRT-PCR) analysis, the synthesized cDNA was amplified in a CFX96 Touch™ Real-Time PCR detection system (Bio-Rad, Hercules, CA) with iQTMSYBR Green Supermix and specific primers (**Supplementary Table S1**). All reactions were performed in triplicate. The relative expression level of each gene was calculated using the ΔΔCt method, as described previously (Livak and Schmittgen, 2001). The *Arabidopsis* *PP2A* (At1g13320) and *Actin8* (At1g49240) genes were used for normalization in the seed and seedling samples, respectively.

### Stomatal Aperture Bioassay

The stomatal aperture bioassay was conducted as described previously, but with some modifications (Lim et al., 2014). Briefly, leaf peels were collected from the rosette leaves of 3-week-old plants and floated on stomatal opening solution [SOS; 50 mM KCl, 10 mM MES-KOH, and 10 μM CaCl_2_ (pH 6.15)]. The peels were incubated at 24°C under fluorescent light for 3 h to obtain >80% stomatal opening in *A. thaliana* Col-0 plants. The buffer was replaced with fresh SOS and leaf peels were further incubated at 4°C under fluorescent light for 3 h. In each individual sample, 100 stomata were randomly observed under a Nikon Eclipse 80i microscope. The widths and lengths of individual stomatal pores were recorded using Image J 1.46r software (http://imagej.nih.gov/ij). Each experiment was performed in triplicate.

## Results

### *RCARs* Are Differentially Regulated in ABA-Deficient Seeds

**Figure 1:**
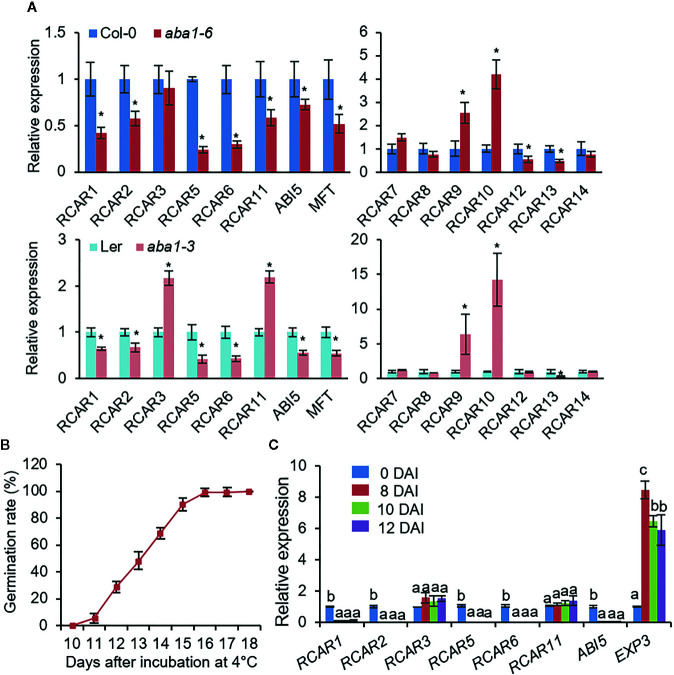
*RCAR* expression in Arabidopsis dry seeds and during seed germination under cold stress conditions. **(A)** Differential *RCAR* expression in dry seeds of ABA-deficient mutants *aba1-6* (Col-0 background; upper) and *aba1-3* (Ler background; bottom). *PP2A* was used as an internal control for normalization. The expression level of each *RCAR* in wild-type (WT) Arabidopsis seeds was set to 1.0. ABA-responsive gene *ABI5* and *MFT* were used as positive controls. **(B)** Germination rate of WT (Col-0) seeds under cold stress (4°C) conditions. For measuring germination rate, 100 seeds were plated on 0.5× MS media, and seeds with radicle protrusion were counted as germinated. **(C)** *RCAR* expression during seed germination under cold stress conditions. ABA-responsive gene *ABI5* and cell wall-related gene *EXP3* were used as controls for seed germination. All data represent mean ± standard deviation (SD) of three independent experiments. Asterisks and different letters indicate significant differences between samples [Student’s *t*-test for **(A)** and ANOVA test for **(C)**, *P* < 0.05].

Several studies based on public microarray data have shown that expression patterns of *RCARs* vary among different tissues and in response to ABA and abiotic stresses, suggesting substantial functional differences among these genes (Kilian et al., 2007; Winter et al., 2007; Park et al., 2009; Santiago et al., 2009; Szostkiewicz et al., 2010; Gonzalez-Guzman et al., 2012; Klepikova et al., 2016). However, *RCAR* gene expression patterns in seeds remain unclear. We investigated expression levels of *RCARs* in dry seeds of *Arabidopsis thaliana* Columbia-0 (Col-0) and Landsberg *erecta* (Ler) ecotypes by quantitative reverse transcription-polymerase chain reaction (qRT-PCR) analysis (**Supplementary Figure S1A**). In seed, the *RCAR* genes were divided into the two groups according to their abundance; transcripts of six *RCARs*—*RCAR1*, *RCAR2*, *RCAR3*, *RCAR5*, *RCAR6*, and *RCAR11*—were relatively abundant, while the rest were less. In this study, we focused on high-abundant *RCAR* genes in this study, of which *RCAR1*, *RCAR2*, *RCAR5*, and *RCAR6* were significantly downregulated after imbibition (**Supplementary Figures S1B, C**). ABA is a key hormone involved in the induction and maintenance of seed dormancy, and its level decreases rapidly following imbibition (Kushiro et al., 2004; Millar et al., 2006). Hence, we postulated that the transcriptional alteration of *RCAR1*, *RCAR2*, *RCAR5*, and *RCAR6* is associated with endogenous ABA level in seeds. We examined expression levels of *RCARs* in the ABA-deficient mutants *aba1-6* (Col-0 background) and *aba1-3* (Ler background). In comparison with wild-type (WT) seeds, ABA-deficient seeds showed upregulated expression of *RCAR9* and *RCAR10*, but downregulated expression of *RCAR1*, *RCAR2*, *RCAR5*, *RCAR6, RCAR12*, and *RCAR13* (**Figure 1A**). Based on these, *RCAR* expression in seeds may be influenced by endogenous ABA level, and downregulation of *RCAR1*, *RCAR2*, *RCAR5*, and *RCAR6* may be associated with the breaking of ABA-induced seed dormancy.

Dormancy is an important adaptive trait that improves plant survival by inhibiting seed germination under unfavorable conditions, including cold stress (Finkelstein et al., 2008). In general, cold stratification (2–5°C for 2–4 days) causes a release of seed dormancy in *Arabidopsis* and promotes seed germination through an increase in the level of endogenous GAs (Baskin and Baskin, 2004; Yamauchi et al., 2004). We wondered how long the germination of the stratified seeds is suppressed under cold stress condition by incubating continuously at low temperature. We sowed freshly harvested seeds of *Arabidopsis* Col-0 on 0.5× MS agar plates and measured the germination rate every day after incubation at 4 ± 1°C in the dark. Seeds started to germinate 10–11 days after incubation (DAI) and all seeds had germinated 16 DAI (**Figure 1B**). In this process, we expected the functional involvement of *RCAR1*, *RCAR2*, *RCAR5*, and *RCAR6* genes. To verify hypothesis, we first examined the expression patterns of *RCARs* during seed germination under cold stress conditions (**Figure 1C**). Genes involved in ABA response and GA-mediated cell wall modification are differentially expressed during seed germination (Liu et al., 2016). We amplified the ABA-responsive gene *ABI5* and the cell wall-related gene *EXPANSIN3* (*EXP3*) as a control; at 8 DAI, transcripts of *ABI5* and *EXP3* were significantly down- and up-regulated, respectively. As expected, expression levels of *RCAR1*, *RCAR2*, *RCAR5*, and *RCAR6* decreased significantly during seed germination, consistent with data obtained using *A. thaliana* Col-0 and Ler ecotypes (**Supplementary Figures S1B, C**); however, we determined no significant change in expression levels of *RCAR3* and *RCAR11* (**Figure 1C**).

### RCAR5 Functions in Delay of Seed Germination Under Prolonged Exposure to Low Temperature

**Figure 2:**
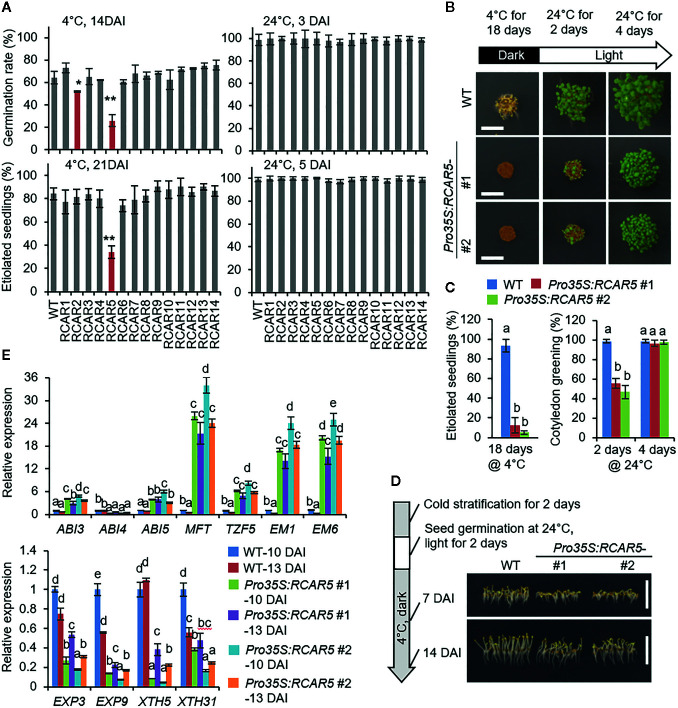
Delayed germination of *Pro35S:RCAR5* seeds under cold stress conditions. **(A)** Germination and seedling growth of *Pro35S:RCAR* mutants under cold stress conditions. For measuring germination rates and seedling growth, 100 seeds of each plant line were plated on 0.5× MS media. After cold stratification, seeds were germinated at 24°C and 4°C in the dark, respectively. The numbers of germinated seeds (with an emerged radicle) and etiolated seedlings were counted at 3 DAI and 5 DAI at 24°C and 14 DAI and 21 DAI 4°C, respectively. **(B, C)** Recovery of delayed germination of *Pro35S:RCAR5* seeds under normal growth conditions. After incubation for 18 days at 4°C in the dark, *Pro35S:RCAR5* seeds were transferred to 24°C with light. For analyzing seedling establishment, 100 seeds of each plant line were plated on 0.5× MS media. The numbers of etiolated seedlings and seedlings with green cotyledon were counted at the indicated time points, and simultaneously representative images were taken. Scale bar = 0.5 cm. **(D)** Post-germinative growth of *Pro35S:RCAR5* mutants under cold stress conditions. After cold stratification for 2 days in the dark, 100 seeds of each plant line were germinated for 2 days at 24°C with light and then incubated again at 4°C in the dark. Representative images were taken at the indicated time points. Scale bar = 1 cm. **(E)** Expression patterns of ABA-responsive genes and cell-wall related genes in the seeds of *Pro35S:RCAR5* mutants and WT plants during germination at 4°C in the dark. All data represent mean ± SD of three independent experiments. Asterisks and different letters indicate significant differences compared with WT [Student’s *t*-test for **(A)** and ANOVA test for **(C, E)**; *P < 0.05; **P < 0.01].

We generated *Arabidopsis* transgenic plants overexpressing each *RCAR* under the control of the 35S promoter. In comparison with WT plants, the 14 transgenic lines showed high expression levels of each *RCAR* gene (>30- to 300-fold) (**Supplementary Figure S2A**) and also high sensitivity to ABA during seed germination and seedling growth (**Supplementary Figures S2B–D**). To investigate how this ABA hypersensitivity affects seed germination under cold stress conditions, we measured the germination rates at 24 and 4°C in the dark after cold stratification (at 4 ± 1°C for 2 days). In normal growth condition, seed germination and seedling growth did not differ significantly between transgenic and WT plants (**Figure 2A**; **Supplementary Figures S2B–E**). However, the germination rates of transgenic lines *Pro35S:RCAR2 and Pro35S:RCAR5* at 14 DAI were significantly lower (56.0% and 25.9%, respectively) than those of WT plants (64.4%) at 4°C (**Figure 2A**). At 21 DAI, the number of etiolated seedlings was significantly lower in *Pro35S:RCAR5* line than in WT and *Pro35S:RCAR2* line, but there was no statistical difference between WT and *Pro35S:RCAR2* line. To determine whether it may be due to a loss of seed viability in the *Pro35S:RCAR5* line, seeds of *Pro35S:RCAR5* (#1) and *Pro35S:RCAR5* (*#2*) were incubated at 4 ± 1°C for 18 days and transferred into normal growth condition (24°C). After 4 days, all seeds germinated and normally developed into seedlings (**Figures 2B, C**). We also wondered if post-germinative growth of *Pro35S:RCAR5* is affected under cold stress conditions. Seeds of *Pro35S:RCAR5* mutants and WT were uniformly germinated for 2 days at 24°C, light after cold stratification and then were incubated 4 ± 1°C for 14 days. As shown in **Figure 2D**, the seedling growth of *Pro35S:RCAR5* mutants was shown to be inhibited early at 4°C compared to that of WT, suggesting that overexpression of the *RCAR5* gene can arrest both pre-germinative and post-germinative growth under cold stress conditions.

To investigate delayed germination of *Pro35S:RCAR5* seeds at the molecular level, we examined expression levels of ABA-responsive genes during germination at 4°C. For isolating RNAs, dry seeds of *Pro35S:RCAR5* and WT lines and their imbibed seeds at 10 DAI (not germinated) and 13 DAI (>50% germinated) were harvested. In dry seeds, overexpression of *RCAR5* gene led to high expression of the ABA-responsive genes *ABSCISIC AICD-INSENSITIVE (ABI) 3*, *ABI4*, *ABI5*, *MOTHER OF FT AND TFL1* (*MFT*), *TANDEM CCCH ZINC FINGER PROTEIN 5* (*TZF5*), *EARLY METHIONINE-LABELED (EM) 1*, and *EM6*, relative to WT seeds (**Supplementary Figure S3A**). However, we determined no significant difference in the expression levels of the *XERICO, NCED6* and *NCED9* genes—which are involved in ABA biosynthesis in seeds (Lefebvre et al., 2006; Zentella et al., 2007; Piskurewicz et al., 2008)—between both plant lines. Consistently, the ABA contents of freshly harvested dry seeds from those plants were also quite similar (**Supplementary Figure S3C**). During germination at 4°C, expression levels of all the ABA-responsive genes, not ABI4, were significantly higher in *Pro35S:RCAR5* seeds than in WT seeds at all time points examined. In contrast, expression of cell wall-related genes—*EXPANSIN (EXP) 3*, *EXP9*, *XYLOGLUCAN ENDOTRANSGLUCOSYLASE/HYDROLASE* (*XTH) 5*, and *XTH31*—was downregulated in *Pro35S:RCAR5* seeds (**Figure 2E**).

**Figure 3:**
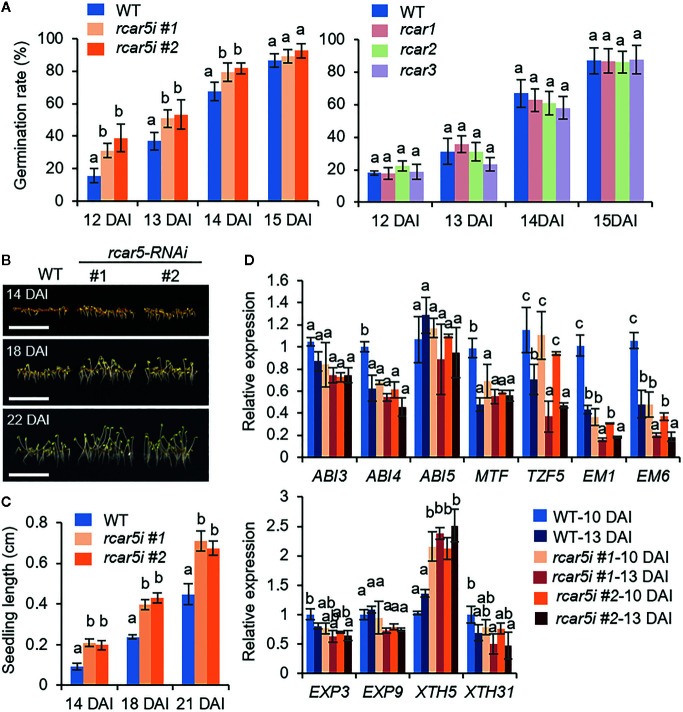
Enhanced germination of *rcar5-*RNAi seeds under cold stress conditions. **(A)** Germination rates of *rcar5*-RNAi mutants, *RCAR* knockout mutants, and WT plants under cold stress conditions. Germination rates of each line (n = 100 seeds) were determined by counting seeds with as emerged radicle from 12 to 15 DAI. **(B, C)** Seedling growth of *rcar5*-RNAi mutants and WT plants under cold stress conditions. Representative images were taken at 18 DAI **(B)**, and seedling lengths of each plant line were measured at the indicated time points **(C)**. Scale bar = 1 cm. **(D)** Expression patterns of ABA-responsive genes and cell-wall related genes in the seeds of *rcar5*-RNAi mutants and WT plants during germination at 4°C in the dark. All data represent mean ± SD of three independent experiments. Different letters indicate significant differences compared with WT (ANOVA test; *P* < 0.05).

Next, we generated transgenic *rcar5*-RNAi (*rcar5i*) mutants because T-DNA insertional mutant of *RCAR5* gene was not available at the Arabidopsis Biological Resource Center. As shown in **Supplementary Figure S4A**, the expression level of *RCAR5* gene, not the other *RCAR* genes, decreased >85% in the seeds of *rcar5i* lines compared to WT seeds. However, RNAi knockdown of *RCAR5* genes did not affect expression of the ABA-responsive genes in dry seeds (**Supplementary Figure S3B**). There was also no alteration between WT and *rcar5i* lines under normal conditions in terms of seed germination, seedling growth, and even ABA sensitivity (**Supplementary Figures S4B–E**). However, the germination rate of *rcar5i* seeds at 4°C was significantly high up to 14 DAI, compared to that of WT (**Figure 3A**), and *rcar5i* seedlings were longer than those of WT (**Figures 3B, C**). In contrast to *rcar5i*, mutants of *RCAR1*, *RCAR2*, *RCAR3*, and *RCAR4* knockout did not affect seed germination at 4°C (**Figure 3A**). Of the ABA-responsive genes and cell wall-related genes, *EM1*, *EM6*, and *XTH5* genes showed consistent patterns with enhanced germination of *rcar5i* lines at 4°C; the expression levels of *EM1*, and *EM6* genes were lower in *rcar5i* seeds than in WT seeds during germination, while *XTH5* expression was significantly higher (**Figure 3D**). Our data suggest that *RCAR5* can function in ABA-mediated inhibition of seed germination under cold stress conditions.

### Cold Stress-Induced Germination Delay of *Pro35S:RCAR5* Seeds Occurs *via* an ABA-Dependent Pathway

**Figure 4:**
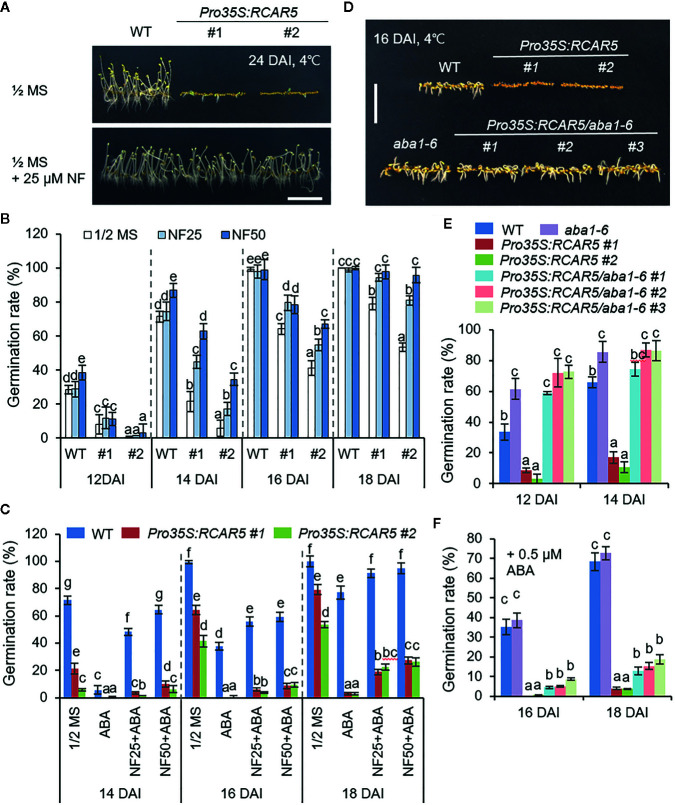
Cold-induced germination delay of *Pro35S:RCAR5* seeds in an ABA-dependent manner. **(A–C)** Effect of norflurazon on seed germination of WT and *Pro35S:RCAR5* lines. Seeds of *Pro35S:RCAR5* and WT were germinated on 0.5× MS medium supplemented with norflurazon (25 or 50 μM) and/or ABA (0.75 μM) and vertically grown at 4°C in the dark. After 24 days, representative images were taken (A). For measuring germination rates of each plant line, the numbers of seeds with emerged radicles were counted at the indicated time points **(B, C)**. **(D–G)** Germination and seedling growth of *Pro35S:RCAR/aba1-6* mutants under cold stress conditions. Seeds of each transgenic line were plated on 0.5× MS medium supplemented with 0 or 0.75 μM ABA and germinated in the dark at 4°C. Representative images were taken at 16 DAI **(D)**. Germination rates **(E, F)** of each plant line were measured at the indicated time points All data represent mean ± SD of three independent experiments, each evaluating 100 seeds of each plant line. Different letters indicate significant differences compared with WT (ANOVA; *P*7 < 0.05). Scale bar = 1 cm.

High expression levels of ABA-responsive genes were shown in *Pro35S:RCAR5* dry seeds and germinated seeds at 4°C (**Figure 2E** and **Supplementary Figure S3**); hence, we postulated that *RCAR5* overexpression delays seed germination at 4°C *via* an ABA-dependent pathway. WT and *Pro35S:RCAR5* seeds were placed on 0.5× MS medium supplemented with norflurazon (NF)—an indirect ABA biosynthesis inhibitor—and germinated at 4°C in the dark. Consistent with the previous study (Piskurewicz et al., 2008), supplementation with NF increased the germination rate in a dose-dependent manner; notably, at 14 DAI, the germination rate of *Pro35S:RCAR5* seeds was 2–6 fold higher in the presence than in the absence of NF (**Figures 4A, B**). This enhanced germination was observed at all the investigated time points, except 12 DAI. There was not any effect of NF on the germination of WT seeds after 16 DAI. In contrast to NF, ABA strongly inhibited seed germination and caused a 4-day delay in the germination of WT seeds (**Figure 4C**). At 18 DAI, 77.1% of WT seeds were germinated on 0.5× MS medium supplemented with 0.75 μM ABA, whereas <3% in *Pro35S:RCAR5* seeds. As predicted, NF was antagonistic to ABA-mediated inhibition of seed germination. In the presence of NF, 48.2–64.5% of WT seeds were germinated at 14 DAI, compared to 5.6% in the absence of NF. NF also improved the germination of *Pro35S:RCAR5* seeds by up to 26%, despite the presence of ABA. To verify the germination delay of *Pro35S:RCAR5* seeds using genetic analysis, we overexpressed *RCAR5* in the ABA-deficient mutant *aba1-6* (*Pro35S:RCAR5/aba1-6*; **Supplementary Figure S5A**) and analyzed the relationship between RCAR5-mediated ABA signaling and delayed seed germination under cold stress conditions. At 4°C, the germination rate of *aba1-6* seeds was 1.3–1.9-fold higher than that of WT seeds; moreover, germinated *aba1-6* seedlings had longer radicles than WT seedlings (**Figures 4D, E**). *RCAR5* overexpression did not alter the germination rate of *aba1-6* mutants at 4°C. However, similar to *Pro35S:RCAR5* seedlings, *Pro35S:RCAR5/aba1-6* seedlings were more sensitive to ABA—as measured by lower seed germination rates and shorter radicles—than were *aba1-6* seedlings (**Supplementary Figures S5B–E**). Owing to this ABA hypersensitivity, the application of exogenous ABA strongly inhibited the germination of *Pro35S:RCAR5/aba1-6* seeds under cold stress conditions (**Figure 4F**). Our data suggest that RCAR5 contributes to the cold stress-induced delay of seed germination *via* an ABA-dependent pathway.

### HAB1, OST1, and ABI5 Are Involved in Delayed Germination of *Pro35S:RCAR5* Seeds Under Cold Stress Conditions

**Figure 5:**
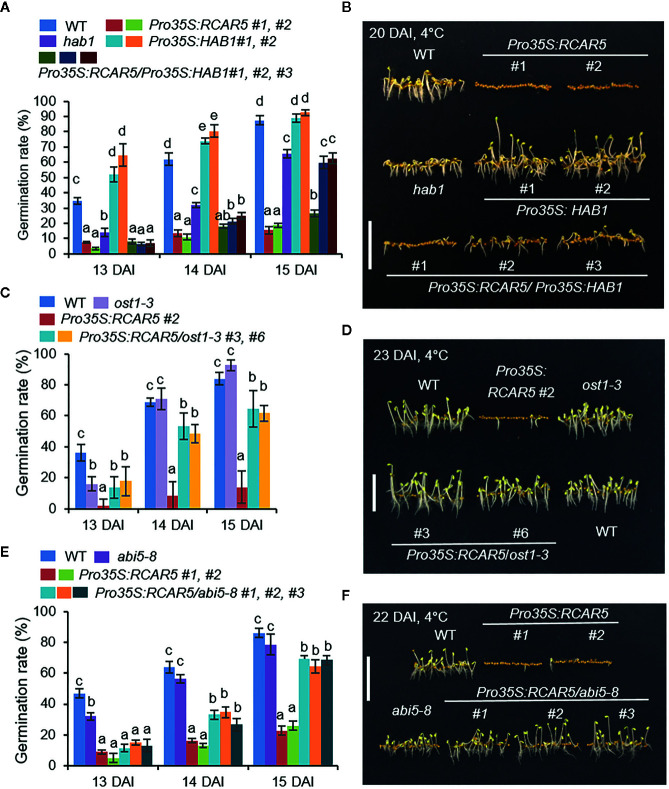
Delayed germination of *Pro35S:RCAR5* seeds under cold stress conditions is regulated by downstream component HAB1, OST1, and ABI5. **(A, B)** Germination and seedling growth of *Pro35S:RCAR5*, *hab1*, *Pro35S:HAB1*, *Pro35S:RCAR5/Pro35S:HAB1*, and WT plants under cold stress conditions. **(C, D)** Germination and seedling growth of *Pro35S:RCAR5*, *ost1-3*, *Pro35S:RCAR5/ost1-3*, and WT plants under cold stress conditions. **(E, F)** Germination and seedling growth of *Pro35S:RCAR5*, *abi5-8*, *Pro35S:RCAR5/abi5-8*, and WT plants under cold stress conditions. Seeds were plated on 0.5× MS medium and germinated in the dark at 4°C. For measuring germination rates, the numbers of seeds with emerged radicles were counted at the indicated time points **(A, C,** **E)**, and representative images were taken **(B, D,** **F)**. Data represent mean ± SD of three independent experiments, each evaluating 100 seeds of each plant line. Different letters indicate significant differences compared with WT (ANOVA; *P* < 0.05). Scale bar = 1 cm.

To examine the mechanism whereby germination of *Pro35S:RCAR5* seeds is delayed under cold stress conditions, we genetically analyzed the downstream components of RCAR5 in ABA signaling. Our previous study has reported the interaction of RCAR5 with HAB1 (Lim and Lee, 2015). We also found that PP2CA can be the interacting partner of RCAR5 through yeast two-hybrid and bimolecular fluorescence complementation assay (BiFC) analyses (**Supplementary Figures S6A, B**). RCAR5–HAB1 interaction occurred in the nucleus and cytoplasm, whereas RCAR5–PP2CA interaction occurred only in the nucleus. The expression levels of *HAB1* and *PP2CA* decreased in seeds after stratification or imbibition (**Supplementary Figures S6C, D**) and increased in cold stress-treated seedlings (**Supplementary Figures S6E–G**). Loss-of-function mutants of *HAB1* and *PP2CA* showed ABA hypersensitivity during seed germination and seedling growth (**Supplementary Figure S6H**) (Saez et al., 2004; Lim et al., 2014). Consistently, under cold stress conditions, seed germination and seedling growth were more strongly inhibited in *hab1* and *pp2ca* mutants than in WT plants; however, at 16 DAI, the germination rates of *hab1* and *pp2ca* mutants were similar to those of WT plants (**Supplementary Figures S6H, I**). In contrast to *hab1*, *Pro35S:HAB1* was hyposensitive to ABA and *Pro35S:RCAR5/Pro35S:HAB1* showed ABA sensitivity similar to *Pro35S:HAB1* (**Supplementary Figure S7**). Using these mutant lines, we examined the functional relationship between *HAB1* and *RCAR5* during seed germination at 4°C. In contrast to *hab1* seeds, *Pro35S:HAB1* seeds germinated more rapidly than WT seeds up to 14 DAI (**Figures 5A, B**). The germination rate of *Pro35S:RCAR5/Pro35S:HAB1* seeds did not differ significantly from that of *Pro35S:RCAR5* seeds at 13 DAI, but then increased close to the germination rate of *hab1* at 15 DAI. This delayed germination of *Pro35S:RCAR5/Pro35S:HAB1* led to stunted seedling growth, relative to other plant lines. Our data suggest that RCAR5-mediated germination inhibition at 4°C is modulated by HAB1 and may be derived in part from differences in ABA sensitivity.

HAB1 and PP2CA can also interact with OST1/SRK2E/SnRK2.6 (hereafter called OST1) involved in the control of seed development and dormancy, together with SRK2D/SnRK2.2 and SRK2I/SnRK2.3 (Nakashima et al., 2009). Under cold stress conditions, seedling growth of a loss-of-function mutant of *OST1*, *ost1-3* (*srk2e*; Yoshida et al., 2002) was not significantly different from those of WT plants at all time points, except 13 DAI (**Figures 5C, D**), consistent with their ABA sensitivity during germination and seedling growth (**Supplementary Figures S8A–E**; Waadt et al., 2015). To examine whether OST1 is required for RCAR5-mediated germination delay under cold stress conditions, we overexpressed *RCAR5* gene in *ost1-3* mutant background (**Supplementary Figure S8A**). The germination rates of *Pro35S:RCAR5/ost1-3* plant lines at 4°C were much higher than that of *Pro35S:RCAR5* and slightly lower than those of WT and *ost1-3* mutant after 14 DAI (**Figure 5C**). Moreover, *Pro35S:RCAR5/ost1-3* displayed seedling growth similar to WT and *ost1-3* mutant at 23 DAI (**Figure 5D**).

Next, we used the ABA-insensitive *abi5* mutant (*abi5-8*; Zheng et al., 2012). As a bZIP transcription factor, ABI5 is mainly expressed in dry seeds and plays a positive role in ABA signaling during seed germination and early seedling growth (Finkelstein and Lynch, 2000; Lopez-Molina et al., 2001). *abi5* mutants showed ABA hyposensitivity (Zheng et al., 2012), whereas *ABI5*-overexpressing plants were hypersensitive to ABA (Lopez-Molina et al., 2001). We overexpressed *RCAR5* in *abi5-8* (*Pro35S:RCAR5/abi5-8*) mutants; ABA sensitivity—as measured by germination rate and seedling establishment—of these mutants was similar to that of *abi5* mutants (**Supplementary Figures S5F–J**). Based on the observed ABA hyposensitivity, we predicted increased germination of *abi5-8* and *Pro35S:RCAR5/abi5-8* seeds under cold stress conditions. However, the germination rate of *abi5* seeds was lower than that of WT seeds only at 13 DAI, after which it was not statistically significant; moreover, at 22 DAI, *abi5-8* seedlings were smaller than WT seedlings (**Figures 5E, F**). *Pro35S:RCAR5/abi5-8* seed germination at 4°C was not different from that of *Pro35S:RCAR5* lines at 13 DAI; however, after 15 DAI the germination rates and seedling growth were almost similar to those of WT seeds. Our data suggest that cold stress-induced delay in the germination of the *Pro35S:RCAR5* lines is regulated by HAB1, OST1, and ABI5.

### *RCAR5* Is Induced in Leaves in Response to Cold Stress in an ABA-Independent Manner

**Figure 6:**
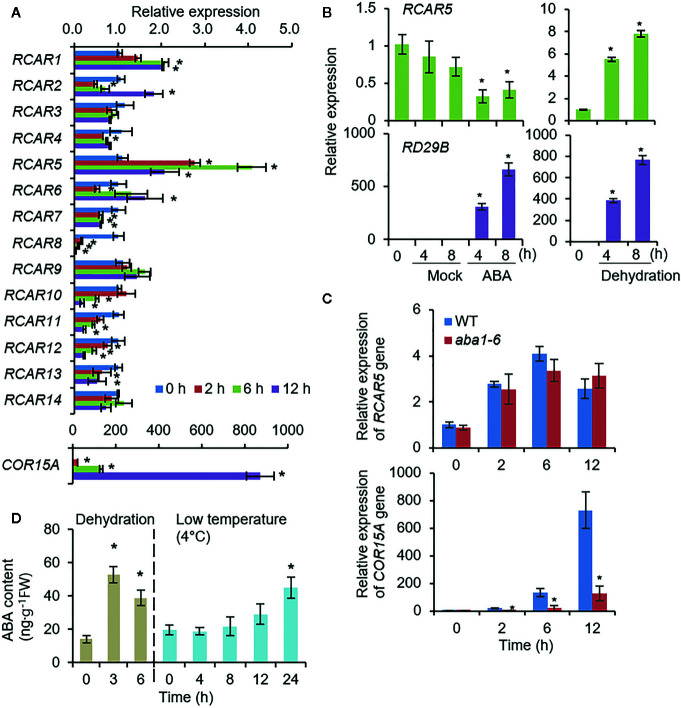
ABA-independent *RCAR5* expression in Arabidopsis leaves in response to cold stress. **(A)** *RCAR* expression in response to cold stress. Three-week-old Arabidopsis Col-0 plants were exposed to 4°C and rosette leaves were harvested at the indicated time points. *Actin8* was used as an internal control for normalization, and *COR15A* was used as a positive control for cold stress treatment. The expression level of each gene at 0 h was set to 1.0. **(B)** *RCAR5* expression pattern in the Arabidopsis leaves after ABA treatment and dehydration stress. *RD29B* was used as a positive control for each treatment. **(C)** *RCAR5* expression pattern in ABA-deficient mutant *aba1-6* in response to cold stress. **(D)** ABA contents in the Arabidopsis leaves after dehydration stress and cold stress treatment. Data represent mean ± SD of three independent experiments. Asterisks indicate significant differences compared with non-treated control (Student’s *t*-test; *P* < 0.05).

To investigate the functional role of RCARs in leaves in response to cold stress, we analyzed expression patterns of *RCARs* in *Arabidopsis* leaves in response to cold stress (4°C). In the eFP browser (Winter et al., 2007), several *RCARs* were responsive to cold stress; however, there was no data available for *RCAR4*, *RCAR5*, *RCAR6*, or *RCAR7* with much lower expression levels in the leaves than the other *RCARs* (**Supplementary Figures S9A–C**). To verify cold stress-induced alteration of *RCAR* gene expression, we performed qRT-PCR analysis. Consistent with in-silico data analysis, expression levels of *RCAR7*, *RCAR8*, *RCAR10*, *RCAR11*, *RCAR12*, and *RCAR13* gradually decreased in response to cold stress, whereas the expression levels of *RCAR1*, *RCAR2*, *RCAR5*, and *RCAR6* were highly induced (>2-fold) relative to the control (**Figure 6A**). Especially, *RCAR5* showed significant alteration (up to 4-fold). The expression level of *RCAR5* in WT leaves was not significantly altered by ABA deficiency (**Supplementary Figure S9D**), but decreased by ABA treatment (**Figure 6B**). Based on these, we wondered whether cold stress-induced *RCAR5* expression occurs in an ABA-independent manner. To confirm this, we analyzed the expression pattern of *RCAR5* in the ABA-deficient mutant *aba1-6* under cold stress conditions. Similar to the WT, *aba1-6* showed a gradual expression of *RCAR5* in rosette leaves in response to cold stress, but *COR15A* induction was significantly lower in *aba1-6* than in WT (**Figure 6C**). Interestingly, ABA did not accumulate within the tested time points by cold treatment, although dehydration treatment significantly increased ABA early. (**Figure 6D**). Our findings suggest that *RCAR5* is induced in *Arabidopsis* leaves by cold stress *via* an ABA-independent pathway.

### *Pro35S:RCAR5* Transgenic Plants Exhibit Enhanced Stomatal Closure in Response to Cold Stress

**Figure 7:**
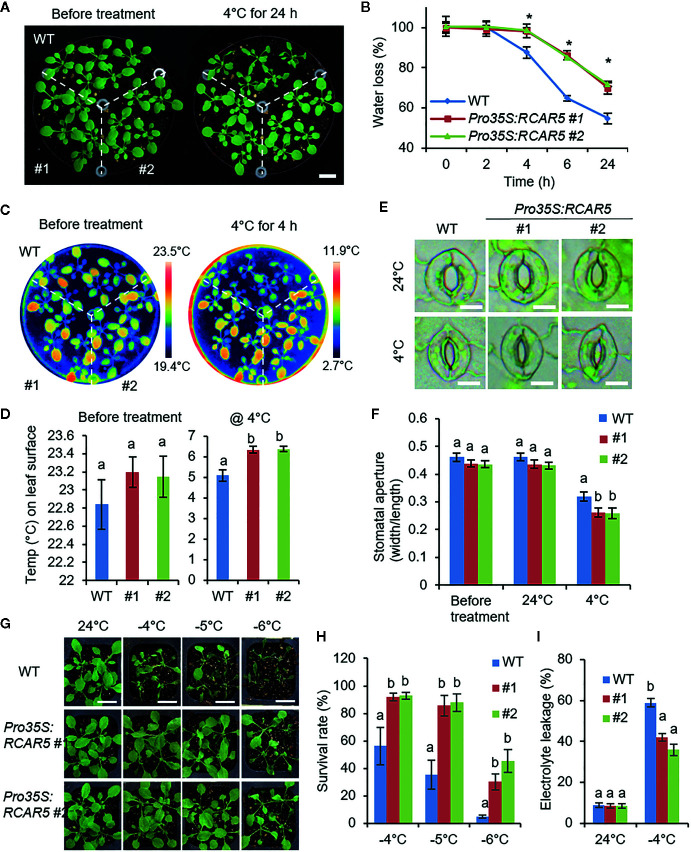
Enhanced tolerance of *Pro35S:RCAR5* plants to cold stress. **(A, B)** Cold stress-induced dehydration of *Pro35S:RCAR5* transgenic lines #1 and #2 and WT plants. Three-week-old Arabidopsis plants were treated with cold stress (4°C) for 24 h, and representative images were taken **(A)**. Scale bar = 1 cm. The fresh weights of each plant line (n = 30) were measured at the indicated time points after cold stress **(B)**. **(C, D)** Leaf temperatures of *Pro35S:RCAR5* plants in response to cold stress. Representative thermographic images of *Pro35S:RCAR5* and WT plants 4 h after cold stress treatment **(C)**; the mean leaf temperatures of the two largest leaves were measured using 20 plants of each line **(D)**. **(E, F)** Stomatal apertures in *Pro35S:RCAR5* transgenic lines and WT plants treated with cold stress. Leaf peels harvested from 3-week-old plants of each line were incubated in chilled stomatal opening solution (SOS) for 2 h at 4°C. Representative images were taken **(E)** and the stomatal apertures were measured under the microscope **(F)**. Scale bar = 10 μm. **(G, H)** Freezing tolerance of *Pro35S:RCAR5* and WT plants. Three-week-old seedlings of each plant line were exposed to freezing temperatures as indicated. After recovery at 24°C for 2 days, representative images were taken **(G)**, and the survival rate of each line was counted **(H)**. Scale bar = 2 cm. **(I)** Electrolyte leakage of *Pro35S:RCAR5* and WT plants. For freezing treatment, 3-week-old seedlings were exposed to −4°C for 1 h. All data represent mean ± SD of three independent experiments. Asterisks and different letters indicate significant differences between WT and transgenic plants [Student’s t-test for **(B)** and ANOVA test for **(D, F, H, I)** *P* < 0.05].

Cold stress triggers leaf wilting and stomatal closure (Wilkinson et al., 2001; Ruelland and Zachowski, 2010). *RCAR5* expression was strongly induced by dehydration, not ABA (**Figure 6B**). Hence, *RCAR5* expression could be triggered by cold stress-induced leaf dehydration in an ABA-independent manner. In comparison with WT plants, *Pro35S:RCAR5* plants showed enhanced tolerance to dehydration stress *via* decreased transpirational water loss from the leaves (**Supplementary Figures S9E–G**) and increased ABA-induced stomatal closure (Lim and Lee, 2015). Based on these, we conducted phenotypic analysis of *Pro35S:RCAR5* plants in response to cold stress. After 24 h incubation at 4°C, WT plants were wilted, but *Pro35S:RCAR5* plants seemed to quite similar to non-treated plants (**Figure 7A**). To monitor this cold stress-induced leaf dehydration in a quantitative manner, we harvested aerial parts from WT and *Pro35S:RCAR5* plants at each time point after incubation at 4°C and simultaneously measured their weights (**Figure 7B**). In comparison with WT plants, *Pro35S:RCAR5* plants showed significantly lower water loss after 4 h incubation at 4°C; the fresh weights of WT and *Pro35S:RCAR5* plants at 24 h were decreased by approximately 44 and 29%, respectively. Interestingly, this phenomenon was observed in *Pro35S:RCAR5* plants, not in *Pro35S:RCAR2* or *Pro35S:RCAR3* plants (**Supplementary Figures S10A–C**). To examine whether the decreased water loss exhibited by *Pro35S:RCAR5* plants is derived from the change in stomatal closure, we measured leaf surface temperature and stomatal apertures in response to cold stress. Under normal growth conditions, there were no significant differences between WT and *Pro35S:RCAR5* plants (**Figures 7C–F**). However, after incubation at 4°C, the leaf temperatures of *Pro35S:RCAR5* plants were higher than those of WT plants; after 4 h incubation, the difference in leaf temperature was statistically significant (**Figures 7C, D**; **Supplementary Figures S10D, E**). Consistently, the stomatal apertures of *Pro35S:RCAR5* plants were significantly smaller than those of WT plants (**Figures 7E, F**); in comparison with non-treated plants, the average stomatal apertures of WT and *Pro35S:RCAR5* plants were reduced by 32% and 41%, respectively. Our data suggest that overexpression of *RCAR5* can lead to rapid stomatal closure under cold stress conditions.

### Enhanced Tolerance of *Pro35S:RCAR5* Transgenic Plants to Cold Stress is Accompanied by High Expression Levels of Cold-Responsive Genes

Based on the enhanced stomatal closure triggered by cold stress as well as ABA, we postulated that *Pro35S:RCAR5* plants can have a higher tolerance to freezing stress. We exposed 3-week-old WT and *Pro35S:RCAR5* seedlings to freezing temperature (approximately −4–−6°C) for 1 h. After transfer to 24°C for 2 days, the survival rate of *Pro35S:RCAR5* seedlings was higher than that of WT; in comparison with *Pro35S:RCAR5* seedlings, WT seedlings were severely wilted and did not survive (**Figures 7G, H**). Under cold stress conditions, the cellular membrane is frequently damaged because of cold stress-induced dehydration (Steponkus, 1984; Uemura et al., 1995). The degree of membrane injury in plants can be evaluated by relative electrolyte leakage (Steponkus, 1984). *Pro35S:RCAR5* seedlings showed significantly lower electrolyte leakage than WT plants (**Figure 7I**). Since RNAi-mediated knockdown of *RCAR5* gene led to promoting seed germination at 4°C, we expected the *rcar5i* mutant lines to show the opposite responses to *Pro35S:RCAR5* under cold stress conditions. However, there was no significant difference between *rcar5i* mutants and WT in terms of leaf temperature, water loss, and freezing tolerance (**Supplementary Figures S4F–J**), which may be due to functional redundancy of the *RCAR* genes.

Next, we selected 12 representative genes—*CBF1*–*3* (AT4G25490, AT4G25470, AT4G25480), *RD29A* (AT5G52310), *RD29B* (AT5G52300), *RAB18* (AT5G66400), *DREB2A* (AT5G05410), *COR15A* (AT2G42540), *COR47* (AT1G20440), *RD26* (AT4G27410), and *KIN1*–*2* (AT3G21960, AT5G15970)—associated with cold stress, dehydration, and ABA signaling. We analyzed the expression patterns of these genes in response to cold stress (4°C). With the exception of *DREB2A* and *COR47*, the expression levels of the investigated genes were higher expressed in the rosette leaves of *Pro35S:RCAR5* transgenic lines than in those of WT plants under normal growth conditions. These different expression levels between the two plant lines were still observed after cold stress treatment, although some genes showed opposite patterns at 12 h (**Supplementary Figure S11**). Our results suggest that high levels of CBF genes during the early stages of cold treatment may help trigger the transcription of target genes in *Pro35S:RCAR5* transgenic lines. In contrast, this high induction of stress genes was not shown in *Pro35S:RCAR5/Pro35S:HAB1* and *Pro35S:RCAR5/ost1-3* mutants (**Supplementary Figure S12**). *hab1* and *ost1-3* also showed similar patterns to WT, suggesting that HAB1 and OST1 could be indirectly involved in upregulation of cold, dehydration, and/or ABA-induced genes in *Pro35S:RCAR5.*

### Enhanced Stomatal Closure of *Pro35S:RCAR5* Transgenic Plants to Cold Stress Is Regulated by OST1 and Is Independent of ABA

**Figure 8:**
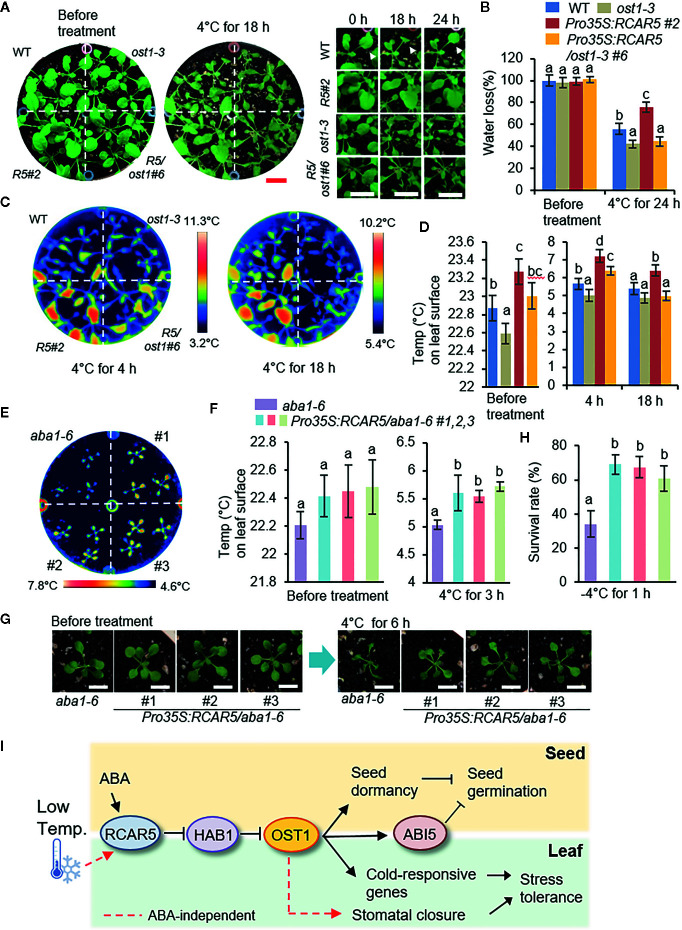
Involvement of OST1 in the enhanced stomatal closure of *Pro35S:RCAR5* plants to cold stress, independent of ABA. **(A, B)** Cold stress-induced dehydration of *Pro35S:RCAR5/ost1-3*, *ost1-3*, and WT plants. Three-week-old seedling of each line were subjected to cold stress (4°C) for 24 h, and representative images were taken at the indicated time points **(A)**. Scale bar = 1 cm. The fresh weights of each plant line (n = 30) were measured at the indicated time points after cold stress **(B)**. Arrowheads indicate leaves that are wilted at 18 h and then recovered at 24 h after cold stress treatments. **(C, D)** Leaf temperatures of *Pro35S:RCAR5/ost1-3*, *ost1-3*, and WT plants in response to cold stress. Representative thermographic images of each plant line at 4 and 18 h after cold stress treatment **(C)**; the mean leaf temperatures of the two largest leaves were measured using 24 plants of each line **(D)**. **(E, F)** Leaf temperatures of *Pro35S:RCAR5/aba1-6* plants in response to cold stress. Representative thermographic images of *Pro35S:RCAR5/aba1-6* and *aba1-6* plants at 3 h after cold stress treatment **(E)**; the mean leaf temperatures of the two largest leaves were measured using 24 plants of each line **(F)**. **(G)** Cold stress-induced dehydration of *Pro35S:RCAR5/aba1-6* and *aba1-6* plants. Three-week-old Arabidopsis plants were subjected to cold stress (4°C) for 6 h, and representative images were taken. Scale bar = 1 cm. **(H)** Freezing tolerance of *Pro35S:RCAR5/aba1-6* and *aba1-6* plants. Three-week-old seedlings of each plant line were exposed to freezing temperatures (−4°C) for 1 h and the survival rate of each line was counted. **(I)** Schematic representation of the functional role of RCAR5 in ABA-mediated seed dormancy and ABA-independent stomatal closure under cold stress conditions. Arrows indicate promotion actions; lines with end bar indicate inhibitory actions. All data represent mean ± SD of three independent experiments. Different letters indicate significant differences between WT and transgenic plants (ANOVA; *P* < 0.05).

ABA is a well-known phytohormone that triggers stomatal closure under abiotic stress conditions such as dehydration, in which OST1 functions as a core signaling component of ABA responses of guard cells (Cutler et al., 2010). To examine whether OST1 is involved in cold stress-induced stomatal closure of *Pro35S:RCAR5* plants, 3-week-old seedlings of WT, *Pro35S:RCAR5*, *ost1-3*, and *Pro35S:RCAR5/ost1-3* were treated at 4°C for 24 h. Although WT and *ost1-3* plants similarly wilted up to 18 h, only some of WT plants recovered when incubated further for 6 h (**Figure 8A**). The fresh weight of *ost1-3* was also significantly lower than that of WT after incubation for 24 h (**Figure 8B**). Compared to WT, the surface temperature in the *ost1-3* leaves was relatively low even under cold stress conditions, but the difference at 18 h became less significant statistically (**Figures 8C, D**). Overexpression of *RCAR5* gene did not confer any difference in *ost1-3* in terms of water loss, leaf temperature, and tolerance under cold stress conditions (**Supplementary Figures S13A–D**); after incubation at 4°C, *Pro35S:RCAR5/ost1-3* plants displayed similar phenotypic responses to *ost1-3.* These data suggest that OST1 functions downstream of RCAR5 in cold-induced stomatal closure.

Next, to examine whether enhanced stomatal closure in *Pro35S:RCAR5* plants to cold stress is associated with ABA, we subjected 3-week-old WT, *Pro35S:RCAR5*, *aba1-6*, and *Pro35S:RCAR5/aba1-6* seedlings to cold stress. In comparison with WT plants, *aba1-6* and *Pro35S:RCAR5/aba1-6* mutant plants wilted rapidly. Thus, it was difficult to distinguish phenotypic differences between *Pro35S:RCAR5/aba1-6* and *aba1-6* mutant plants in response to cold stress conditions; hence, in further studies, we used only *Pro35S:RCAR5/aba1-6* and *aba1-6* as a control. Under normal growth conditions, leaf temperatures were slightly higher in *Pro35S:RCAR5/aba1-6* than in *aba1-6* plants, but the difference was not significant; however, after cold stress treatment (4°C for 3 h), leaf temperatures were significantly higher in *Pro35S:RCAR5/aba1-6* plants than in *aba1-6* plants (**Figures 8E, F**). After a further 3 h of incubation, *Pro35S:RCAR5/aba1-6* plants were less wilted than *aba1-6* plants (**Figure 8G**). Similar to *Pro35S:RCAR5* plants, this physiological characteristic of *Pro35S:RCAR5/aba1-6* plants contributed to enhanced survival rates after freezing treatment (−4°C for 1 h) (**Figure 8H**). However, qRT-PCR analysis revealed that expression levels of the ABA-, dehydration-, and cold-responsive genes *RD29B*, *RAB18*, *KIN1*, *COR47*, and *COR15A* did not differ significantly between *Pro35S:RCAR5/aba1-6* and *aba1-6* plants in response to cold treatment (4°C for 6 h) (**Supplementary Figure S14**), and this may be derived from ABA deficiency. Our data suggest that cold stress induces stomatal closure *via* an ABA-independent pathway involving RCAR5 and OST1.

## Discussion

As ABA receptors, *Arabidopsis* RCAR*s* function redundantly in the regulation of seed germination, stomatal aperture, and transcriptional activation in response to ABA (Park et al., 2009; Gonzalez-Guzman et al., 2012). Nevertheless, many studies have suggested a distinct function of RCARs in ABA and abiotic stress signaling, based on their different expression patterns, biochemical properties, and genetic analyses data (Dupeux et al., 2011; Hao et al., 2011; Gonzalez-Guzman et al., 2012; Antoni et al., 2013; Zhao et al., 2013; Zhao et al., 2014; Zhao et al., 2016). Our data provide new insight into the involvement of RCAR5 in cold stress responses, including the delay of seed germination and rapid stomatal closure *via* ABA-dependent and ABA-independent pathways, respectively (**Figure 8I**).

Owing to the functional redundancy of the *RCAR* gene family, we initially generated transgenic plants overexpressing each of the 14 *RCARs*. Overexpression of these *RCARs* conferred ABA hypersensitivity in terms of seed germination and seedling growth, but the variation of ABA sensitivity in each mutant could be due to the variation of the expression levels of *RCAR*s (**Supplementary Figure S2**). Seed dormancy and germination are influenced by ABA content and ABA sensitivity. Several studies have shown that mutations in ABA biosynthesis and signaling components influence the level of seed dormancy. Seed germination occurs more rapidly in ABA-deficient mutants—such as *aba1* and *aba2*—than in WT plants (Xiong et al., 2001; Gonzalez-Guzman et al., 2002). Dominant-negative mutants of *ABI1* (*abi1-1*) and *ABI2* (*abi2*) and a mutant defective in three *SnRKs* (*snrk2.2*, *snrk2.3*, and *snrk2.6*) showed reduced seed dormancy, consistent with their negative and positive roles in ABA signaling, respectively (Fujita et al., 2009; Nakashima et al., 2009). Hence, we predicted delayed seed germination of *Pro35S:RCAR* mutants under cold stress conditions. Of the fourteen lines, only *Pro35S:RCAR5* and *Pro35S:RCAR2* seeds showed markedly delayed germination under cold stress conditions, compared to WT (**Figure 2A**), but seedling growth of *Pro35S:RCAR2* was almost similar to that of WT at 21 DAI. Consistently, seed germination at 4°C was enhanced in *rcar5*-RNAi, but not *rcar1*, *rcar2*, and *rcar3* (**Figure 3A**), implying RCAR5 plays a crucial role in the cold-induced inhibition of seed germination. Endogenous ABA is likely to inhibit the germination of *Pro35S:RCAR5* seeds under cold stress conditions. *RCAR5*-mediated seed dormancy was blocked by ABA deficiency derived from NF treatment and loss-of-function of *ABA1* (**Figure 4**) and was regulated by HAB1, OST1, and ABI5—downstream components of RCAR5 in ABA signaling (**Figure 5**). In this process, we do not rule out the possibility that other factors involved in ABA signaling participate due to the following two points: (1) ABI5 is shown to be modulated by two other SnRKs, SRK2D/SnRK2.2 and SRK2I/SnRK2.3, as well as OST1 in *Arabidopsis* seeds, (Nakashima et al., 2009). (2) several SnRK2-PP2C interactions can form in ABA signaling (Yoshida et al., 2006; Fujii et al., 2009; Lee et al., 2009; Umezawa et al., 2009; Vlad et al., 2009). Similar to *HAB1* and *PP2CA*, *ABI2* and *AHG1* expression levels decreased in imbibed seeds after cold stratification (**Supplementary Figures S6C, D**).

Relative to other *RCARs*, transcripts of *RCAR1*, *RCAR2*, *RCAR5*, and *RCAR6* accumulated strongly in dry seeds regardless of the transcriptional difference between Col-0 and Ler ecotypes (**Supplementary Figure S1A**). Furthermore, the four *RCARs* were downregulated in ABA-deficient mutant *aba1-6* and during seed germination (**Figures 1A, C**; **Supplementary Figures S1B, C**). Regardless of these similar expression patterns, the effect of gene overexpression on seed germination under cold stress conditions had varied, suggesting that the degree of cold-induced germination delay could be partly associated with ABA sensitivity. This hypothesis can be supported by the results of *Pro35S:RCAR5/Pro35S:HAB1* mutant (**Figures 5A, B**; **Supplementary Figure S7**). Similarly, the discrepancy between ABA sensitivity and seed dormancy was observed in the ABA-insensitive *abi5-8* mutant (**Figures 5E, F**; **Supplementary Figures S5G–I**). ABI5 does not control seed dormancy but does regulate seed germination and seedling growth in response to ABA (Finkelstein and Lynch, 2000; Lopez-Molina et al., 2001). Compared to WT, the germination rate of *abi5-8* seeds at 4°C was lower at 13 DAI, but after 14 DAI not statistically significant (**Figure 5E**), implying that ABI5 plays role in cold-induced seed germination delay. Taken together, the results of *Pro35S:RCAR5*/*abi5-8* suggest that ABI5 functions downstream of RCAR5.

In contrast to the expression pattern in dry seeds, *RCAR5* expression in leaf tissues was much weaker than that of other *RCAR* gene family members and was not influenced by ABA deficiency (**Supplementary Figures S9C, D**). Nevertheless, *RCAR5* expression was significantly upregulated in response to cold stress (**Figure 6A**), which was through an ABA-independent pathway. Consistently, *RCAR5* expression was influenced by dehydration, but not by ABA treatment (**Figure 6B**). Some RCARs—including RCAR1, RCAR3, and RCAR10—inhibit PP2Cs in the absence of ABA (Hao et al., 2011). These raised the possibility that *RCAR5* functions in plant, especially in the leaves, cold stress responses *via* an ABA-independent pathway. In response to cold stress, enhanced stomatal closure was particularly shown in *Pro35S:RCAR5* plants, not in *Pro35S:RCAR2* and *Pro35S:RCAR3* plants (**Figure 7**; **Supplementary Figures S10A–C**). We further showed that leaf temperatures of loss-of-function mutants of *HAB1* and *PP2CA—*which can interact with RCAR5*—*did not differ significantly from those of WT plants in response to cold stress (**Supplementary Figures S13E–G**). However, we cannot rule out the involvement of a pleiotropic effect of *Pro35S:RCAR5* plants and functional redundancy of HAB1 and PP2CA. In contrast, OST1 was needed for rapid stomatal closure of *Pro35S:RCAR5* plants in response to cold stress (**Figures 8A–D**; **Supplementary Figures S13A–D**). OST1 can show ABA-independent activation by environmental stimuli, such as osmotic stress and cold stress, as well as ABA (Mustilli et al., 2002; Yoshida et al., 2006; Ding et al., 2015). Based on these, we postulated that cold-stress induced rapid stomatal closure in *Pro35S:RCAR5* plants could occur *via* an ABA-independent pathway. Consistent with our hypothesis, Wilkinson et al. (2001) have reported rapidly induced stomatal closure in the leaves of *Commelina communis* in response to cold stress, possibly owing to increased uptake of apoplastic calcium, but not ABA, into guard cells. ABA biosynthesis also seems not to be an early event in the cold stress response. In *Arabidopsis*, endogenous ABA accumulated weakly at 6 h, and its level increased up to 4-fold after 15 h exposure to 4°C day/2°C night temperatures (Lång et al., 1994). Moreover, expression of ABA synthesis-related genes was not upregulated at 3, 6, or 24 h after exposure to 0°C (Lee et al., 2005). We also confirmed that there was no change in ABA content from the leaves within 12 h after exposure to 4°C (**Figure 6D**). Here, surface temperature and fresh weight of leaves differed significantly between *Pro35S:RCAR5* and WT plants 4 h after cold treatment. In comparison with the timing of ABA biosynthesis, stomatal closure of *Pro35S:RCAR5* plants seems to start earlier. Consistently, *RCAR5* overexpression in *aba1-6* mutants led to enhanced stomatal closure in response to cold stress. Our data imply that *RCAR5* can be induced in response to cold stress and contributes to cold stress-induced stomatal closure *via* an ABA-independent pathway. Nevertheless, we did not rule out the possibility that ABA is potentially responsible for cold-stress-induced rapid stomatal closure in *Pro35S:RCAR5*, which was due to the following two reasons: (1) ABA content was quantified from the whole leaf, but not guard cells. (2) It is not still clear whether ABA content is changed in local areas of the leaf in response to cold stress. The involvement of ABA in cold stress responses is still controversial. Several studies have suggested that cold stress triggers ABA biosynthesis and that ABA plays an important role in the cold stress response—in particular cold acclimation, as shown in ABA-deficient and ABA-insensitive mutants (Lång et al., 1994; Thomashow, 1999; Xiong et al., 2001; Kushiro et al., 2004). For example, ABA-deficient mutants *aba1* and *aba3* showed impaired freezing tolerance characterized by low expression levels of ABA-responsive genes that may function in cold stress (Xiong et al., 2001). Also, the application of exogenous ABA triggers cold acclimation and enhanced freezing tolerance in *Arabidopsis* (Thomashow, 1999).

In conclusion, our study provides evidence for two different functions of RCAR5 *via* ABA-dependent and ABA-independent pathways in plant tissues such as seeds and leaves. However, the precise function of RCAR5 in the cold stress response remains unclear. Together with transcriptional regulation, studying the protein level of RCAR5 will provide important evidence to understand the functional role of RCAR5 on cold stress. Also, further studies to elucidate the functional interactions RCAR5–HAB1/PP2CA–OST1 in germination delay and rapid stomatal closure under cold stress conditions, and to identify transcription factors that regulate RCAR5 expression *via* ABA-dependent and/or ABA-independent pathways, are required.

## Data Availability Statement

The original contributions presented in the study are included in the article/**Supplementary Material**; further inquiries can be directed to the corresponding author.

## Author Contributions

CWL performed most of the experiments. CWL and SCL designed the experiments, analyzed the data, and wrote the manuscript.

## Funding

This work was supported by a grant from the “Next-Generation BioGreen 21 Program for Agriculture and Technology Development (project no. PJ01316801),” Rural Development Administration, and by the National Research Foundation of Korea (NRF) grant funded by the Korea Government (MSIT) (no. 2018R1A5A1023599, SRC), Republic of Korea.

## Conflict of Interest

The authors declare that the research was conducted in the absence of any commercial or financial relationships that could be construed as a potential conflict of interest.
