# Supplementary material for: ABA-Dependent and ABA-Independent Functions of RCAR5/PYL11 in Response to Cold Stress
Source: Front Plant Sci. 2020 Sep 25;11:587620. doi: 10.3389/fpls.2020.587620 (PMC7545830; doi:10.3389/fpls.2020.587620)
Supplement: Supplementary file 1 [file Table_1.pdf]

TABLE S1. Sequences of primers used in this study.

Primer name	Primer sequence (5'-3')
For qRT-PCR	
Actin8	Forward: CAACTATGTTCTCAGGTATTGCAGA
(At1g49240)	Reverse: GTCATGGAAACGATGTCTCTTTAGT
COR15A	Forward: GATACATTGGGTAAAGAAGCTGAGA
(At2g42540)	Reverse: ACATGAAGAGAGAGGATATGGATCA
DREB2A	Forward: CTACAAAGCCTCAACTACGGAATAC
(At5g05410)	Reverse: AAACTCGGATAGAGAATCAACAGTC
COR47	Forward: TGAAGAGGAAGTGAAGAAAGAAAAA
(At1g20440)	Reverse: AAAATAAAGGATCAAATGCAATCAA
CBF1	Forward: GATGAGGAGACAATGTTTGGGATGC
(At4g25490)	Reverse: GGAAACGACTATCGAATATTAGTAACTC
CBF2	Forward: TCGGTTCAATGGAACTATAATTTTG
(At4g25470)	Reverse: TTACCATTTACATTCGTTTCTCACA
CBF3	Forward: GCGTTTCAGGATGAGATGTGTGATG
(At4g25480)	Reverse: TGTACGGACGGAAGCGGCA
RAB18	Forward: GGAAGAAGGGAATAACACAAAAGAT
(At5g66400)	Reverse: GCGTTACAAACCCTCATTATTTTTA
RD29A	Forward: CACAATCACTTGGCTCCACTGTTG
(At5g52310)	Reverse: ACCTAGTAGCTGGTATGGAGGAACT
RD29B	Forward: GTTGAAGAGTCTCCACAATCACTTG
(At5g52300)	Reverse: ATACAAATCCCCAAACTGAATAACA
RD26	Forward: AGGTCTTAATCCAATTCCAGAGCTA
(At4g27410)	Reverse: ACCCATCAGTAACTTCACATCTCTC
KIN1	Forward: TGTTAACTTCGTGAAGGACAAGAC
(At5g15960)	Reverse: AAGTTTGGCTCGTCTAATAATTTTG
KIN2	Forward: TGTTAACTTCGTGAAGGACAAGAC
(At5g15970)	Reverse: ACAACAACAAGTACGATGAGTACGA
RCAR1	Forward: AGACTTGCTACTTTGTTGAAGCACT
(At1g01360)	Reverse: ACAAATAACAAACCACCAGGAGATA
RCAR2	Forward: GAGATGTACGGAGCTCTAGTGACGG
(At4g01026)	Reverse: TCCAATCTCTCTGTACTGGTGGTTGC
RCAR3	Forward: ACACGACAGAATCGAGAGTCTAAAG
(At5g53160)	Reverse: CACATGAAATTAAAAAGAGGGTTTG
RCAR4	Forward: CAAAGAAGGTGGAGAGCGAGTACAT
(AT4G27920)	Reverse: CCACTTCTCTTACGCTACCAACCTC
RCAR5	Forward: ATGGAAACTTCTCAAAAATATCATACG
(At5g45860)	Reverse: CTCTCTCGGCTGAACTCCGCTG
RCAR6	Forward: TGAAAACAT CTCAAGAACA GCATGTATG
(At5g45870)	Reverse: CTCGCCGGAAGATCGGAAAC
RCAR7	Forward: AGTCTCACGTGATGGTGGTAAGTAT
(At4g18620)	Reverse: TGAGCTTAGCAAGTGAAGTAAGGTT
RCAR8	Forward: GAGGAAACTCTAAGCTTCGTTGATA
(At5g05440)	Reverse: AGGTACTCGAAATTTTCTTCCTTGT
RCAR9	Forward: GTACAAACACTTCGTGAAAAGCTG
(At2g40330)	Reverse: GACTTGTAGTTCATGAGTCTGTGGT
RCAR10	Forward: GAGAGCAAGAAGAAGATGTCTCTGT
(At2g38310)	Reverse: TCGGTTCTTTATGGGATATGTAAAA
RCAR11	Forward: CTGATACGGTTGTGAAGCTTAATTT
(At4g17870)	Reverse: GCAAAAACAGACAAAAGAAGAAGAG
RCAR12	Forward: GACGGTTCATAGATTTGAGAAAGAA
(At5g46790)	Reverse: CCAAATTTCATCATTACCTAACCTG
RCAR13	Forward: ATATACCACAAGGTAACACGGAAGA
(At1g73000)	Reverse: CTAAGCCCATTATAGCCCATAGTTT
RCAR14	Forward: GTCAGAGAAGTGACCGTAATCTCC
(At2g26040)	Reverse: TTTTAGTGTCTTCCTCTGTGTTTCC

Table S1. (continued)

Primer name	Primer sequence (5'-3')
For qRT-PCR	
HAB1	Forward: GACTACCTCTCAATGCTTGCTCTAC
(in CDS+3' UTR)	Reverse: CCCTTCTAACAATATTGAGTAATTAAC
HAB1 (in CDS)	Forward: TAGAAAATGCTGGAGGCAAAGTT
(At1g72770)	Reverse: TCAGGTTCTGGTCTTGAACTTTCTTT
PP2CA	Forward: ACTTGAGGAAGAGGAGGAATAATCA
(At3g11410)	Reverse: CCAGCCTGAATTAAGAGCTAACTAA
ABI3	Forward: TCATAGTCATATACTCCGACGTCAA
(At3G24650)	Reverse: TGCCCTCTTTCTTATTTGGTACATA
ABI4	Forward: ATTCCAACACCAACAGTATCAGAAT
(At2G40220)	Reverse: GTTCAAATCCTCCATCGAACTATTA
ABI5	Forward: CAGTGGAGAAAGTAGTGGAGAGAAG
(At2G36270)	Reverse: CCCTTGACTTCAAACTCTCAAAATA
TZF5	Forward: CAGAGTAGTCCTCTACGATTTGCTC
(At5G44260)	Reverse: GGGAAAGATCTCAAGTAGTTCACAG
MFT	Forward: CACCGTTACATATTGGTACTTTTCC
(At1G18100)	Reverse: ATAGGTAAAGGTTCCTTTCGGTTTT
EM1	Forward: AGGACTCAGTACGATGGAAAAATCT
(At3G51810)	Reverse: GCGCTACATTAGACCCTAGTTAAAA
EM6	Forward: AGTTAGGAACTGAAGGATATCAGCA
(At2G40170)	Reverse: GTACTGGAAAACAAACGAGACACTT
XERICO	Forward: GACAATGAGTGTTCTGTGTGTTTGT
(At2G04240)	Reverse: ACCTATACAGTGAAAAGCAGTCACC
XTH5	Forward: CACCATCTACAACTATTGCACTGAC
(At5G13870)	Reverse: TGAATTCATATTCTCGGTTTCACTT
XTH31	Forward: ACCATTTGTGGCTAAGTACAAAAAC
(At3G44990)	Reverse: TTGTTTGTTTGTTTTAACATTCTGG
EXP3	Forward: ATCTTGTTCTGGTAACTAACGTTGC
(At2G37640)	Reverse: TCAGATTTCAGACTCGAAAGTTTTT
EXP9	Forward: AATCTCTTTCTTTCCGAGTCAAAAC
(At5G02260)	Reverse: CCCAATTCCAAATCGTAAACTAAAT
NCED6	Forward: CATGCAACACTCTCTT CGTTCTGA
(At3G24220)	Reverse: ACTCAATCTTGTCGAGCATCGTAG
NCED9	Forward: TCAAGAATCGACTATGGCTTCTACT
(At1G78390)	Reverse: TGACATCGTTACATAGTTTCTTCCA
PP2A	Forward: TATCGGATGACGATTCTTCGTGCAG
(At1G13320)	Reverse: GCTTGGTCGACTATCGGAATGAGAG
For cloning	
RCAR1	Forward: ATGATGGACGGCGTTGAAGG
	Reverse: TCACTGAGTAATGTCCTGAGAAGCCA
RCAR2	Forward: ATGGAGATGATCGGAGGAGACG
	Reverse: TCAAAGGTTGGTTTCTGTATGATTCT
RCAR3	Forward: ATGGAAGCTAACGGGATTGAG
	Reverse: TTAGACTCTCGATTCTGTCGTGTCTTG
RCAR4	Forward: ATGAACGGTGACGAAACAAAG
	Reverse: TCATATCTTCTTCTCCATAGATTCTG
RCAR5	Forward: ATGGAAACTTCTCAAAAATATCATAC
	Reverse: TTACAACTTTAGATGAGCCACCCT
RCAR6	Forward: ATGAAAACATCTCAAGAACAGCAT
	Reverse: TTAAGTGAGCTCCATCATCTTCTC
RCAR7	Forward: ATGGAAAGTTCTAAGCAAAAACG
	Reverse: TTACTTCATCATTTTCTTTGTGAGC
RCAR8	Forward: ATGAGGTCACCGGTGCAACTC
	Reverse: TTATTGCCGGTTGGTACTTCG

Table S1. (continued)

Primer name	Primer sequence (5'-3')
For cloning	
RCAR9	Forward: ATGCCAACGTCGATACAGTTTCAG
	Reverse: TTACGAGAATTTAGAAGTGTTCTCGG
RCAR10	Forward: ATGCTTGCCGTTCACCGTCC
	Reverse: TCACAGAGACATCTTCTTCTTGCTCTCA
RCAR11	Forward: ATGCCTTCGGAGTTAACACCAGA
	Reverse: TCACGTCACCTGAGAACCACTTCC
RCAR12	Forward: ATGGCGAATTCAGAGTCCTCCT
	Reverse: TTACCTAACCTGAGAAGAGTTGTTGTT
RCAR13	Forward: ATGAATCTTGCTCCAATCCATGAT
	Reverse: TCAGGTCGGAGAAGCCGTGG
RCAR14	Forward: ATGAGCTCATCCCCGGCCGT
	Reverse: TTATTCATCATCATGCATAGGTGCAG
RCAR5-RNAi	Forward: ATGGAAACTTCTCAAAAATATCATACG
	Reverse: CTCTCTCGGCTGAACTCCGCTG
