# Supplementary material for: ABA-Dependent and ABA-Independent Functions of RCAR5/PYL11 in Response to Cold Stress
Source: Front Plant Sci. 2020 Sep 25;11:587620. doi: 10.3389/fpls.2020.587620 (PMC7545830; doi:10.3389/fpls.2020.587620)
Supplement: Supplementary file 2 [file Image_1.pdf]

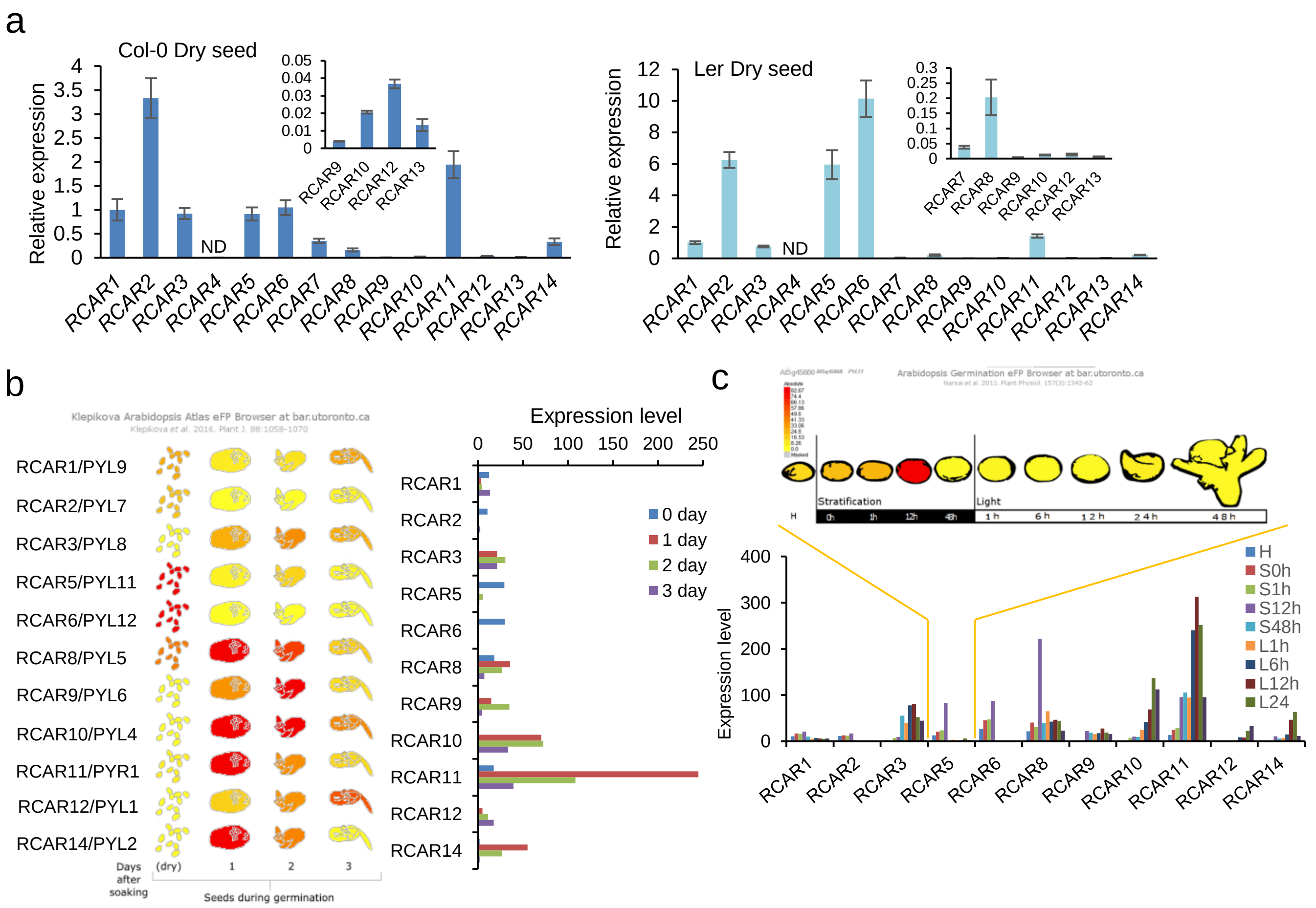

FIGURE S1 Expression patterns of *RCAR* genes during seed germination. (a) Relative expression levels of *RCAR* genes in dry seeds of Arabidopsis ecotype Col-0 (upper) and Ler (bottom). *Actin8* was used as an internal control for normalization and the expression level of *RCAR1* from each ecotype was set to 1.0. (b, c) Relative expression levels of *RCAR* genes during germination. Data were obtained from the Arabidopsis eFP Browser with 'Germination' (b) (Winter et al., 2007) and 'Klepikova Atlas' (c) (Klepikova et al., 2016) as data sources in the Bio-Analytic Resource for Plant Biology (http://bar.utoronto.ca/efp/cgi-bin/efpWeb.cgi). H, seeds from the silique; S, stratification; L, light.
