# Supplementary material for: ABA-Dependent and ABA-Independent Functions of RCAR5/PYL11 in Response to Cold Stress
Source: Front Plant Sci. 2020 Sep 25;11:587620. doi: 10.3389/fpls.2020.587620 (PMC7545830; doi:10.3389/fpls.2020.587620)
Supplement: Supplementary file 3 [file Image_2.pdf]

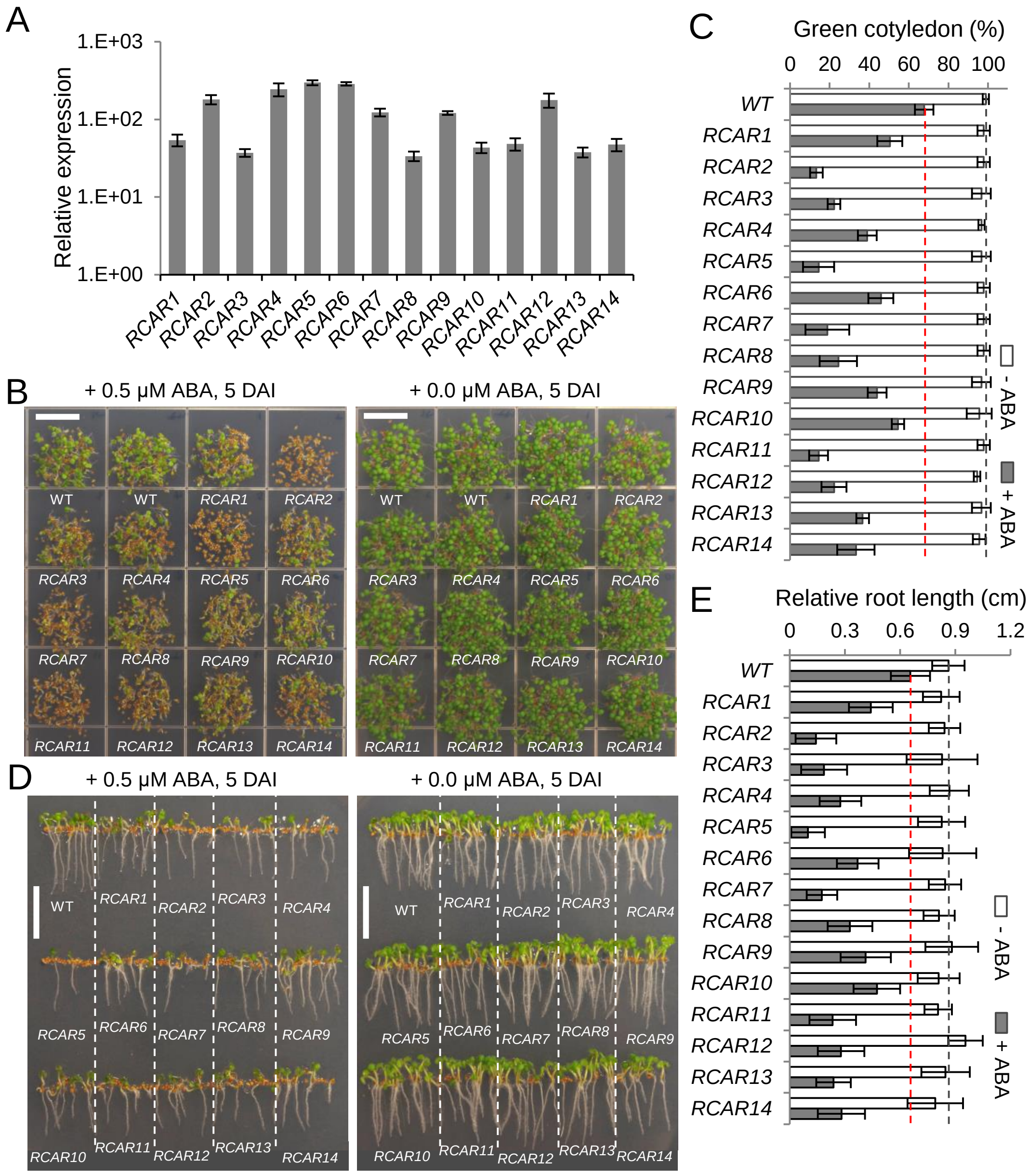

FIGURE S2 Enhanced ABA sensitivity of *Pro35S:RCAR* transgenic plants during seed germination and seedling growth. (A) Expression levels of *RCAR* genes in the leaves of *Pro35S:RCAR* transgenic plants. *Actin8* was used as an internal control for normalization. The relative expression level of each gene in mutants was calculated by setting that of WT to 1.0. (B-E) Seedling development of *Pro35S:RCAR* transgenic lines and WT plants in the presence of ABA. Seeds of *Pro35S:RCAR* transgenic lines were germinated on 0.5× MS medium supplemented with 0 μM or 0.5 μM ABA and grown at 24°C in the light. At 5 days after incubation (DAI), cotyledon greening (C) and root length (E) were measured and representative images were taken (B,D). Data represent mean ± SD of three independent experiments, each evaluating 100 seeds of each plant line. Scale bar = 1cm.
