# Supplementary material for: ABA-Dependent and ABA-Independent Functions of RCAR5/PYL11 in Response to Cold Stress
Source: Front Plant Sci. 2020 Sep 25;11:587620. doi: 10.3389/fpls.2020.587620 (PMC7545830; doi:10.3389/fpls.2020.587620)
Supplement: Supplementary file 4 [file Image_3.pdf]

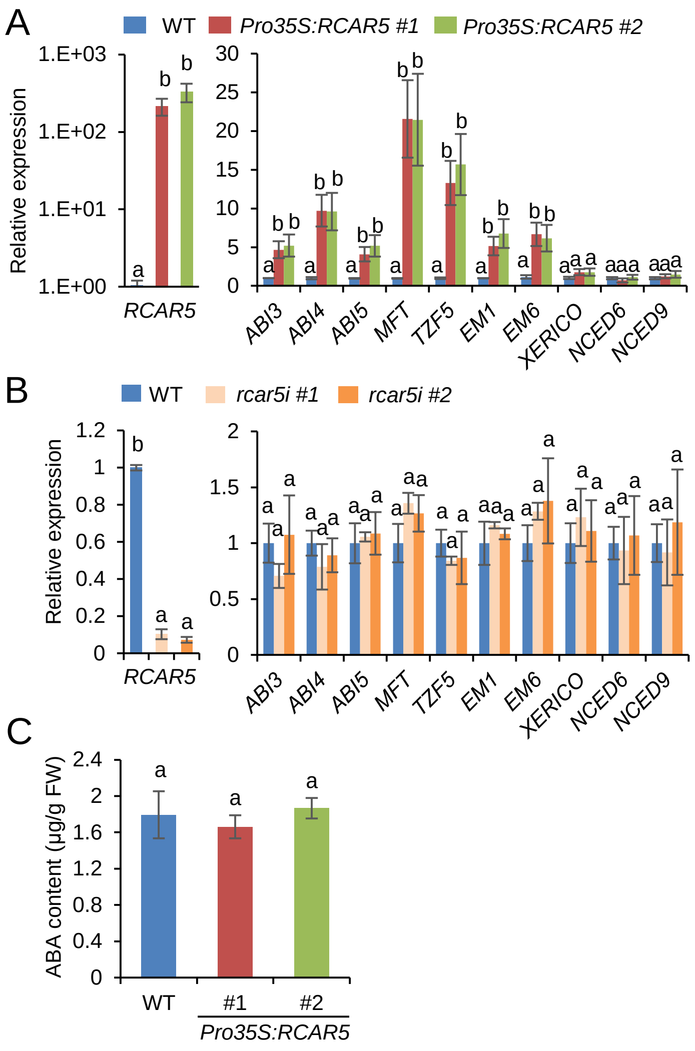

FIGURE S3 ABA-responsive genes and ABA quantification in Arabidopsis dry seeds. (A, B) Expression patterns of ABA-responsive genes in dry seeds of *Pro35S:RCAR5* (a) and *RCAR5-RNAi* (b) mutants. *PP2A* was used as an internal control for normalization. The expression level of each gene in WT was set to 1.0. (C) Quantification of ABA contents in dry seeds of *Pro35S:RCAR5* mutants and Col-0 plants. ABA content was measured in freshly harvested dry seeds of each plant lines by using the Phytodetek-ABA kit (Agdia Inc., Elkhart, IN, USA). FW, Fresh weight. All data represent mean ± SD of three independent experiments. Different letters indicate a significant difference between WT and transgenic plants (ANOVA; $P < 0.05$)..
