# Supplementary material for: ABA-Dependent and ABA-Independent Functions of RCAR5/PYL11 in Response to Cold Stress
Source: Front Plant Sci. 2020 Sep 25;11:587620. doi: 10.3389/fpls.2020.587620 (PMC7545830; doi:10.3389/fpls.2020.587620)
Supplement: Supplementary file 5 [file Image_4.pdf]

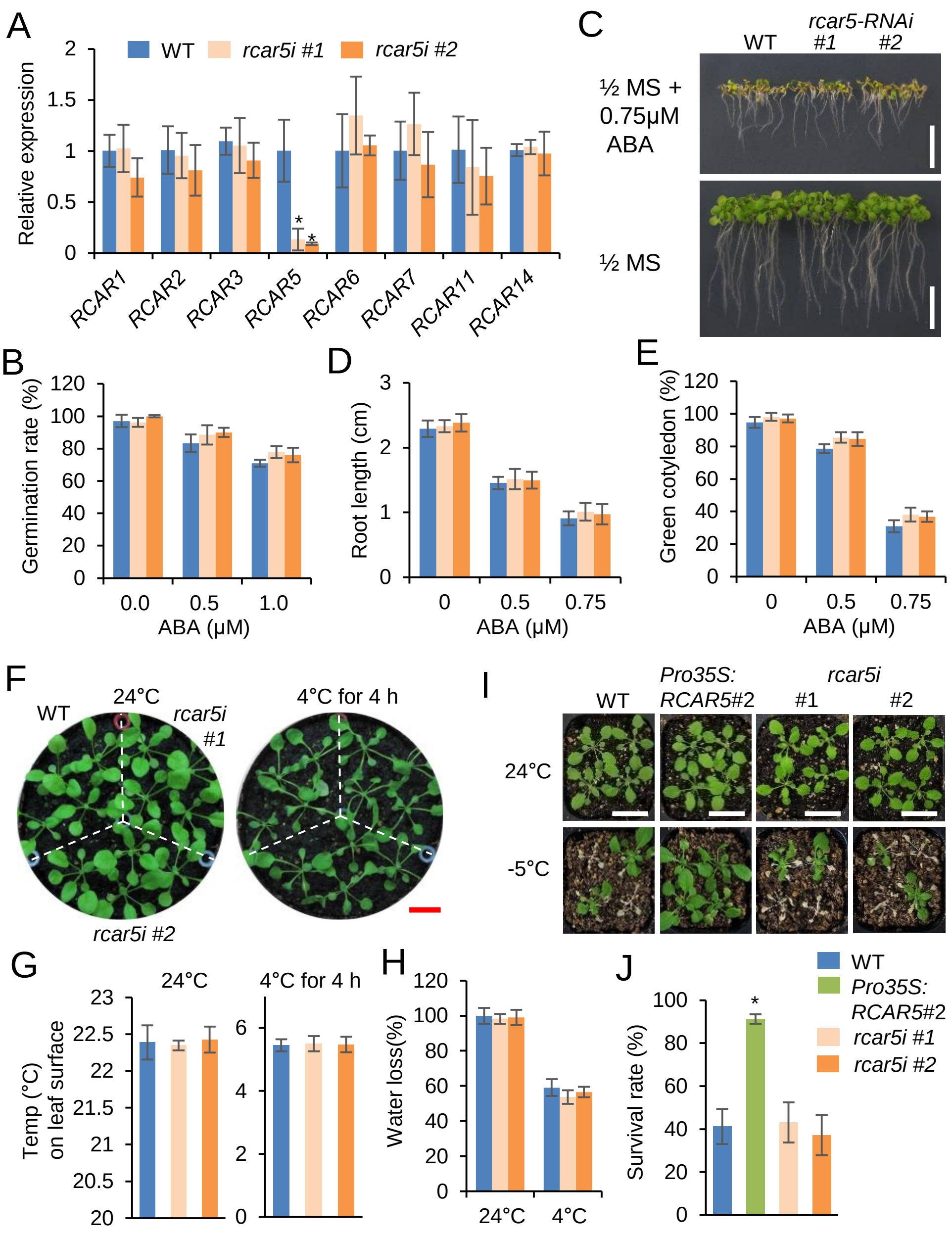

A
Relative expression
WT
rcar5i #1
rcar5i #2
RCAR1
RCAR2
RCAR3
RCAR5
RCAR6
RCAR7
RCAR11
RCAR14
C
WT
rcar5-RNAi
#1
#2
½ MS + 0.75μM ABA
½ MS
B
Germination rate (%)
ABA (μM)
D
Root length (cm)
ABA (μM)
E
Green cotyledon (%)
ABA (μM)
F
WT
24°C
rcar5i #1
rcar5i #2
4°C for 4 h
I
WT
Pro35S: RCAR5#2
rcar5i
#1
#2
24°C
-5°C
G
24°C
4°C for 4 h
Temp (°C) on leaf surface
H
Water loss(%)
24°C
4°C
J
Survival rate (%)
WT
Pro35S: RCAR5#2
rcar5i #1
rcar5i #2

Figure S4

FIGURE S4 ABA sensitivity of *RCAR5-RNAi* mutants during seed germination and seedling growth. (A) Expression levels of *RCAR* genes in the seeds of *RCAR5-RNAi* mutants. *PP2A* was used as an internal control for normalization, and the expression level of each *RCAR* in WT plants was set to 1.0. (B) Germination rates of *RCAR5-RNAi* mutants and WT plants on 0.5× MS medium supplemented with 0 μM, 0.5 μM, or 1.0 μM ABA. The numbers of seeds with emerged radicles were counted 3 days after plating. (n=100 per plant line) (C-E) Seedling development of *RCAR5-RNAi* mutants and WT plants in the presence of ABA. Seeds of each plant line were germinated on 0.5× MS medium supplemented with 0 μM, 0.5 μM, or 0.75 μM ABA and vertically grown at 24°C in the light. At 7 days after incubation (DAI), root length (D) and cotyledon greening (E) were measured and representative images were taken (C). (F, G) Phenotypic response of *RCAR5-RNAi* mutants in response to cold stress. Three-week-old Arabidopsis plants (n=32) were exposed to 4°C for 4 h and representative images were taken (F). Scale bar= 1 cm. At the same time, the mean leaf temperatures of the two largest leaves were measured using 20 plants of each line (G). (H) Water loss from *RCAR5-RNAi* mutants after cold stress treatment. The fresh weights of each plant line (n=30) were measured 24 h after treatment. (I, J) Freezing tolerance of *RCAR5-RNAi* mutants. Three-week-old seedlings of *RCAR5-RNAi* mutants and WT plants (n=32) were exposed to -5°C for 1 h and representative images were taken (I). Scale bar= 2 cm. After recovery at 24°C for 2 days, the survival rate of each line was counted (J). All data represent mean ± SD of three independent experiments. Asterisks indicate significant differences between WT and *RCAR5-RNAi* mutants (ANOVA; $P < 0.05$).
