# Supplementary material for: ABA-Dependent and ABA-Independent Functions of RCAR5/PYL11 in Response to Cold Stress
Source: Front Plant Sci. 2020 Sep 25;11:587620. doi: 10.3389/fpls.2020.587620 (PMC7545830; doi:10.3389/fpls.2020.587620)
Supplement: Supplementary file 6 [file Image_5.pdf]

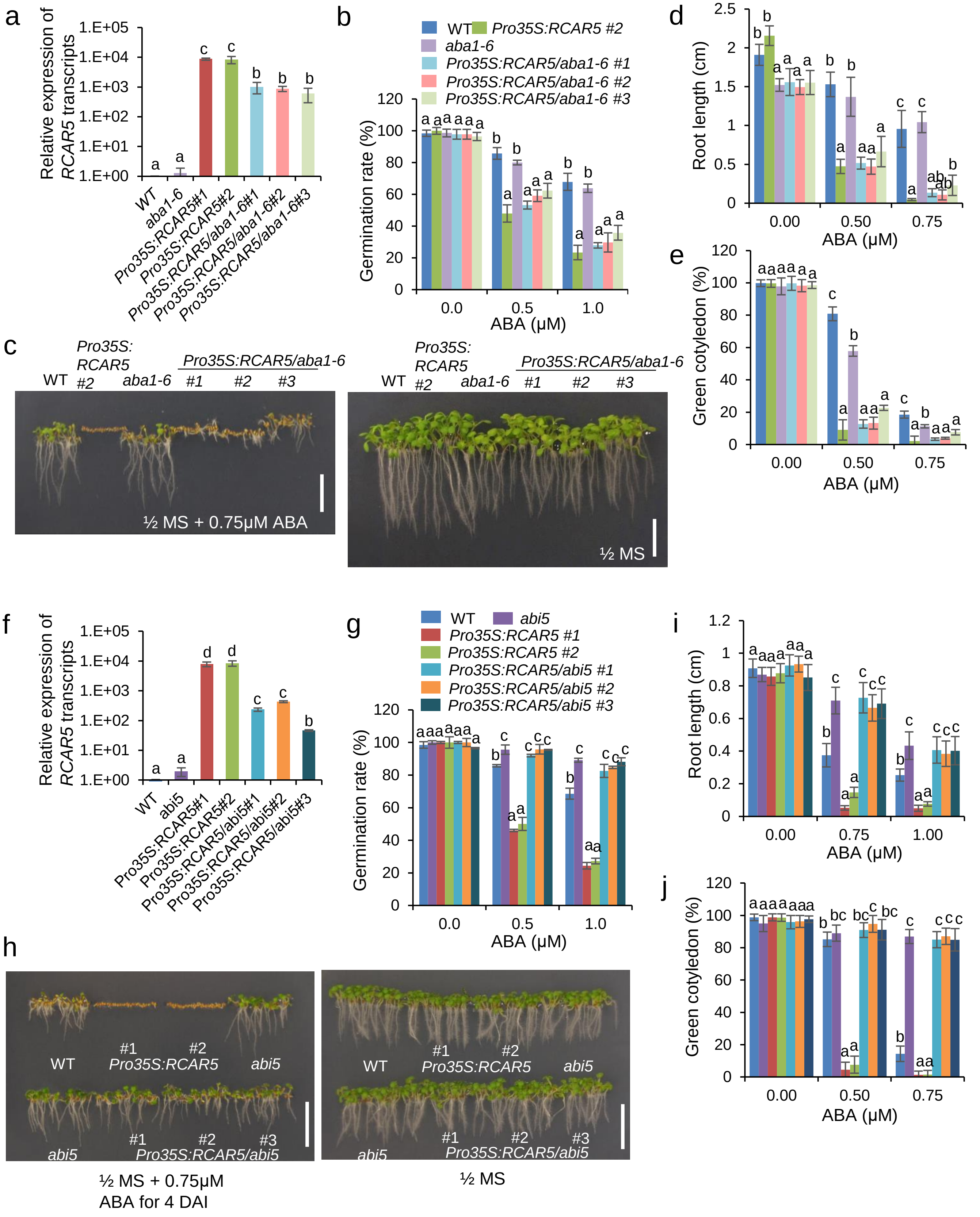
a
Relative expression of RCAR5 transcripts
1.E+05
1.E+04
1.E+03
1.E+02
1.E+01
1.E+00
WT
aba1-6
Pro35S:RCAR5#1
Pro35S:RCAR5#2
Pro35S:RCAR5/aba1-6#1
Pro35S:RCAR5/aba1-6#2
Pro35S:RCAR5/aba1-6#3
b
WT
Pro35S:RCAR5 #2
aba1-6
Pro35S:RCAR5/aba1-6 #1
Pro35S:RCAR5/aba1-6 #2
Pro35S:RCAR5/aba1-6 #3
Germination rate (%)
ABA (μM)
d
Root length (cm)
ABA (μM)
e
Green cotyledon (%)
ABA (μM)
c
Pro35S: RCAR5 #2
Pro35S:RCAR5/aba1-6
WT
aba1-6
#1
#2
#3
½ MS + 0.75μM ABA
½ MS
f
Relative expression of RCAR5 transcripts
WT
abi5
Pro35S:RCAR5#1
Pro35S:RCAR5#2
Pro35S:RCAR5/abi5#1
Pro35S:RCAR5/abi5#2
Pro35S:RCAR5/abi5#3
g
WT
abi5
Pro35S:RCAR5 #1
Pro35S:RCAR5 #2
Pro35S:RCAR5/abi5 #1
Pro35S:RCAR5/abi5 #2
Pro35S:RCAR5/abi5 #3
Germination rate (%)
ABA (μM)
i
Root length (cm)
ABA (μM)
j
Green cotyledon (%)
ABA (μM)
h
WT
#1
#2
Pro35S:RCAR5
abi5
#3
Pro35S:RCAR5/abi5
½ MS + 0.75μM ABA for 4 DAI
½ MS

Figure S5

FIGURE S5 ABA sensitivity of *aba1-6, abi5, Pro35S:RCAR5/aba1-6,* and *Pro35S:RCAR5/abi5* transgenic plants during seed germination and seedling growth. (a) Expression levels of *RCAR5* gene in the leaves of *Pro35S:RCAR5/aba1-6* transgenic plants. *Actin8* was used as an internal control for normalization and the expression level of *RCAR5* in WT plants was set to 1.0. (b) Germination rates of *Pro35S:RCAR*, *Pro35S:RCAR5/aba1-6*, *aba1-6*, and WT plants on 0.5× MS medium supplemented with 0 μM or 0.5 μM ABA. The numbers of seeds with emerged radicles were counted 3 days after plating. (c-e) Seedling development of *Pro35S:RCAR5*, *Pro35S:RCAR5/aba1-6*, *aba1-6*, and WT plants in the presence of ABA. Seeds of each plant line (n=100) were germinated on 0.5× MS medium supplemented with 0 μM, 0.5 μM, or 0.75 μM ABA and vertically grown at 24°C in the light. At 7 days after incubation (DAI), root length (d) and cotyledon greening (e) were measured and representative images were taken (c). (f) Expression levels of *RCAR5* gene in the leaves of *Pro35S:RCAR5/abi5* transgenic plants. *Actin8* was used as an internal control for normalization and expression level of *RCAR5* in WT was set to 1.0. (g) Germination rates of *Pro35S:RCAR5, Pro35S:RCAR5/abi5*, *abi5,* and WT plants on 0.5× MS medium supplemented with 0 μM or 0.5 μM ABA. The numbers of seeds with emerged radicles were counted 3 days after plating. (h-j) Seedling development of *Pro35S:RCAR5*, *Pro35S:RCAR5/abi5*, *abi5*, and WT plants in the presence of ABA. Seeds of each plant line (n=100) were germinated on 0.5× MS medium supplemented with 0 μM or 0.75 μM ABA and vertically grown at 24°C in the light. At 7 DAI, root length (i) and cotyledon greening (j) were measured and representative images were taken (h). Data represent mean ± SD of three independent experiments. Different letters indicate significant differences between WT and transgenic plants (ANOVA; $P < 0.05$). Scale bar = 1 cm.
