# Supplementary material for: ABA-Dependent and ABA-Independent Functions of RCAR5/PYL11 in Response to Cold Stress
Source: Front Plant Sci. 2020 Sep 25;11:587620. doi: 10.3389/fpls.2020.587620 (PMC7545830; doi:10.3389/fpls.2020.587620)
Supplement: Supplementary file 7 [file Image_6.pdf]

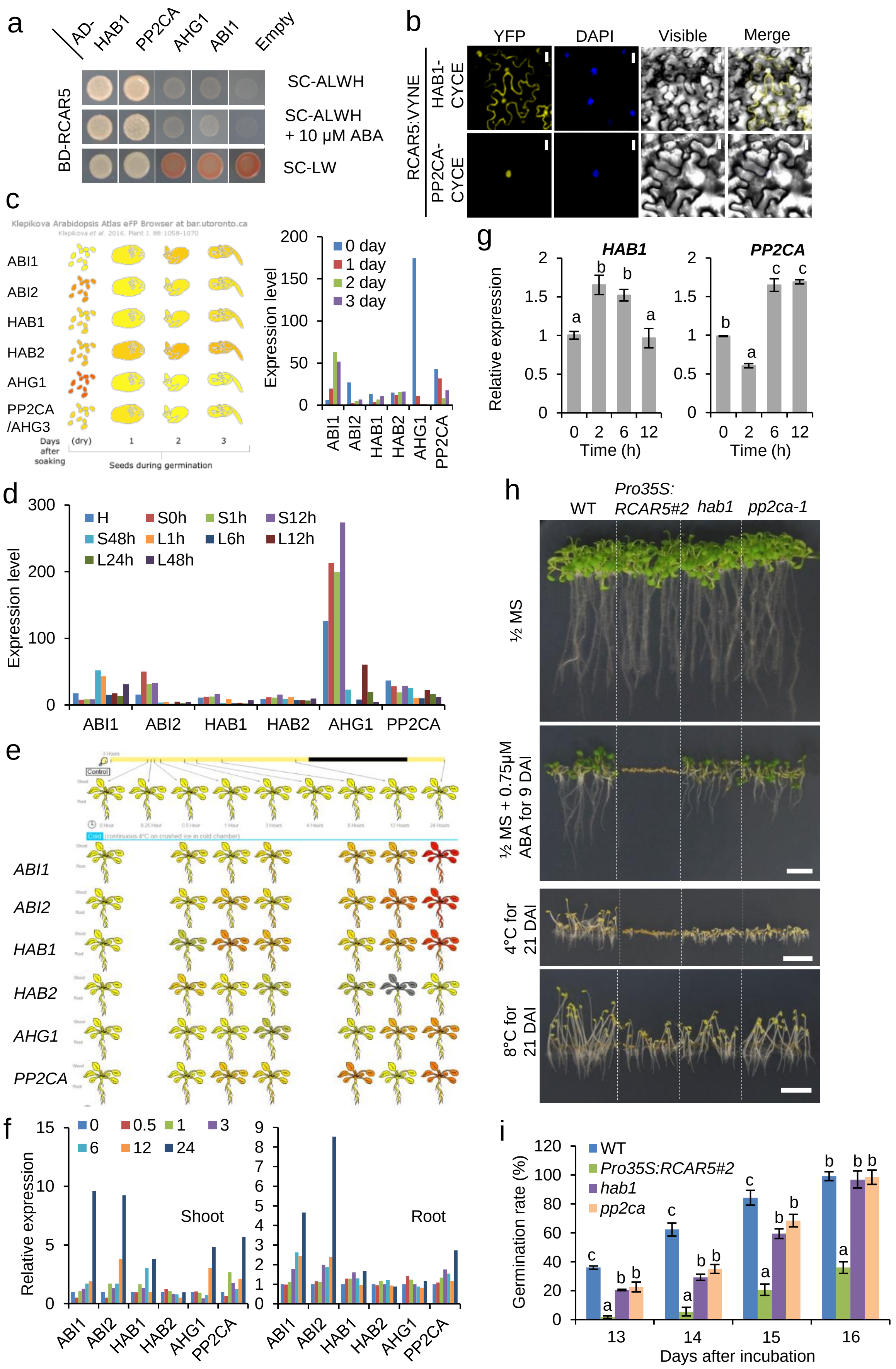

a
AD-
HAB1
PP2CA
AHG1
ABI1
Empty
BD-RCAR5
SC-ALWH
SC-ALWH
+ 10 µM ABA
SC-LW
b
YFP
DAPI
Visible
Merge
RCAR5:VYNE
HAB1-CYCE
PP2CA-CYCE
c
Klepikova Arabidopsis Atlas eFP Browser at bar.utoronto.ca
ABI1
ABI2
HAB1
HAB2
AHG1
PP2CA
/AHG3
Days after soaking
(dry)
1
2
3
Seeds during germination
Expression level
0 day
1 day
2 day
3 day
g
Relative expression
HAB1
PP2CA
Time (h)
d
Expression level
H
S0h
S1h
S12h
S48h
L1h
L6h
L12h
L24h
L48h
ABI1
ABI2
HAB1
HAB2
AHG1
PP2CA
h
WT
Pro35S:
RCAR5#2
hab1
pp2ca-1
½ MS
½ MS + 0.75µM ABA for 9 DAI
4°C for 21 DAI
8°C for 21 DAI
e
Control
Cold
ABI1
ABI2
HAB1
HAB2
AHG1
PP2CA
f
Relative expression
Shoot
Root
0
0.5
1
3
6
12
24
i
Germination rate (%)
WT
Pro35S:RCAR5#2
hab1
pp2ca
Days after incubation

Figure S6

FIGURE S6 Sensitivity of *hab1* and *pp2ca* mutant plants to cold stress. (a) Physical interaction of RCAR5 with HAB1 and PP2CA in yeast. Interaction was indicated by growth on selection medium (SC-Ade-His-Leu-Trp); growth on SC-Leu-Trp was used as a control (right). (b) BiFC assay of interactions of RCAR5 with HAB1 and PP2CA. RCAR5:VYNE was co-expressed with HAB1:CYCE or PP2CA:CYCE in the leaves of tobacco. Scale bar = 20 mm. (c, d) Relative expression levels of *PP2C* genes during germination. Data were obtained from the Arabidopsis eFP Browser with 'Germination' (c) (Winter et al., 2007) and 'Klepikova Atlas' (d) (Klepikova et al., 2016) as data sources in the Bio-Analytic Resource for Plant Biology (http://bar.utoronto.ca/efp/cgi-bin/efpWeb.cgi). H, seeds from the silque; S, stratification; L, light. (e, f) Relative expression levels of *PP2C* genes in Arabidopsis shoots and roots in response to cold stress. Data were obtained from the Arabidopsis eFP Browser with 'Abiotic stress' (Winter et al., 2007) as the data source. (g) qRT-PCR analysis of *HAB1* and *PP2CA* gene expression in Arabidopsis leaves in response to cold stress (4°C). *Actin8* was used as an internal control for normalization. The expression level of each gene at 0 h was set to 1.0. (h) Vertical growth of *Pro35S:RCAR5*, *hab1*, *pp2ca-1*, and WT plants exposed to ABA or LT. Seeds of each plant were plated on 0.5× MS medium supplemented with 0 μM or 0.75 μM ABA and germinated under normal or cold stress conditions. Scale bar = 1 cm. Representative images were taken at 8 DAI (for ABA) and 21 DAI (for LT). (i) Germination rates of *Pro35S:RCAR5*, *hab1*, *pp2ca-1*, and WT plants. Seeds of these plants (n=100) were plated on 0.5× MS medium and germinated in the dark at 4°C. All data represent mean ± SD of three independent experiments. Different letters indicate significant differences between WT and transgenic plants (ANOVA; $P < 0.05$).
