# Supplementary material for: ABA-Dependent and ABA-Independent Functions of RCAR5/PYL11 in Response to Cold Stress
Source: Front Plant Sci. 2020 Sep 25;11:587620. doi: 10.3389/fpls.2020.587620 (PMC7545830; doi:10.3389/fpls.2020.587620)
Supplement: Supplementary file 8 [file Image_7.pdf]

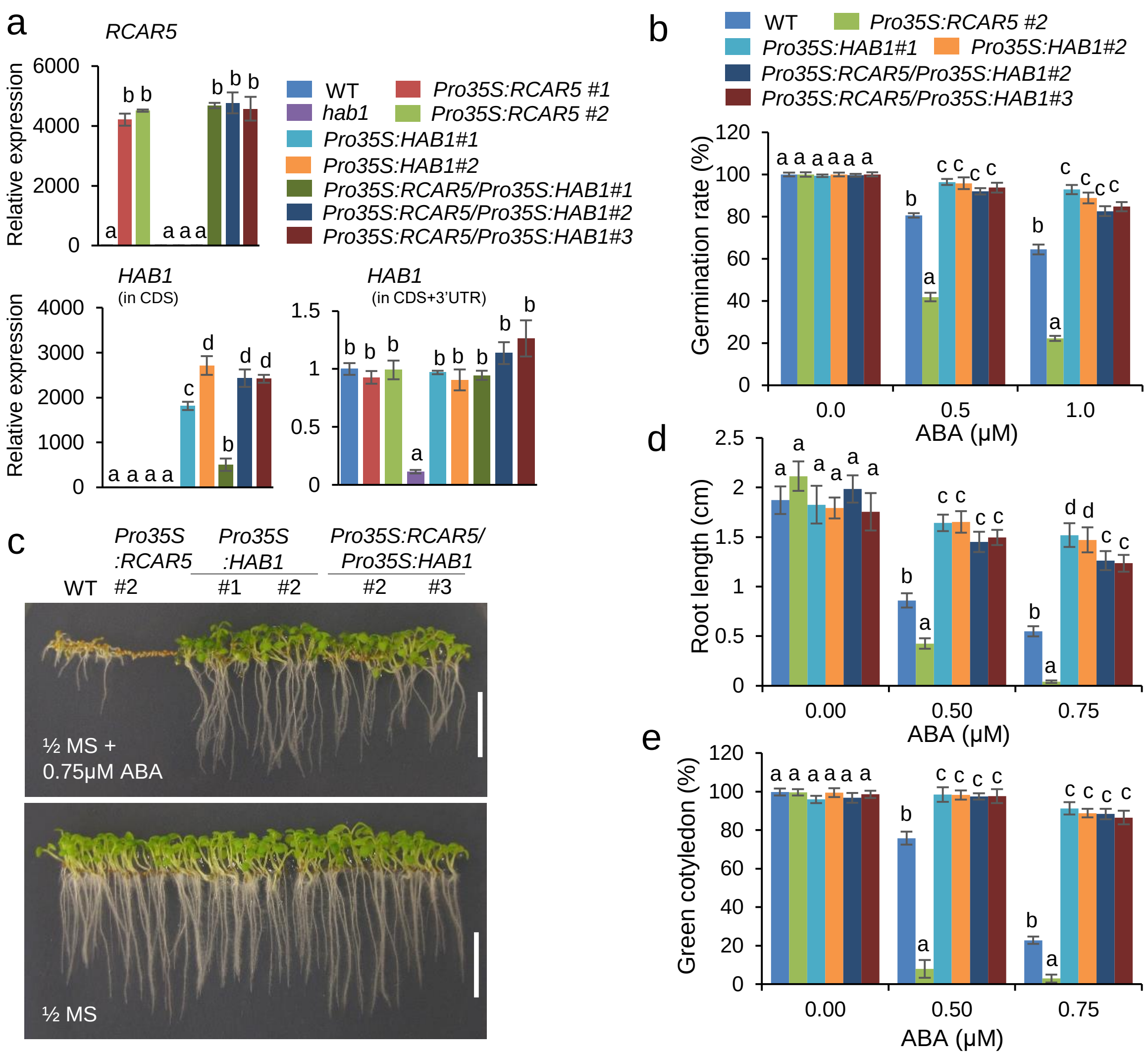

FIGURE S7 Reduced ABA sensitivity of *Pro35S:HAB1* and *Pro35S:RCAR5/HAB1* transgenic plants during seed germination and seedling growth. (a) Expression levels of *RCAR5* and *HAB1* genes in the leaves of *Pro35S:HAB1* and *Pro35S:RCAR5/Pro35S:HAB1* transgenic plants. *Actin8* was used as an internal control for normalization. The expression levels of *RCAR5* or *HAB1* genes in WT plants were set to 1.0. (b) Germination rates of *Pro35S:RCAR5*, *Pro35S:HAB1*, *Pro35S:RCAR5/Pro35S:HAB1*, and WT plants on 0.5× MS medium supplemented with 0 μM, 0.5 μM, or 1 μM ABA. The numbers of seeds with emerged radicles were counted 3 days after plating. (n=100 per plant line) (c–e), Seedling development of *Pro35S:RCAR5*, *Pro35S:HAB1*, *Pro35S:RCAR5/Pro35S:HAB1*, and WT plants in the presence of ABA. Seeds of each plant line were germinated on 0.5× MS medium supplemented with 0 μM, 0.5 μM, or 0.75 μM ABA and vertically grown at 24°C in the light. At 7 DAI, root length (d) and cotyledon greening (e) were measured and representative images were taken (c). All data represent mean ± SD of three independent experiments. Different letters indicate significant differences between WT and transgenic plants(ANOVA; $P < 0.05$). Scale bar = 1 cm.

Figure S7
