# Supplementary material for: ABA-Dependent and ABA-Independent Functions of RCAR5/PYL11 in Response to Cold Stress
Source: Front Plant Sci. 2020 Sep 25;11:587620. doi: 10.3389/fpls.2020.587620 (PMC7545830; doi:10.3389/fpls.2020.587620)
Supplement: Supplementary file 10 [file Image_9.pdf]

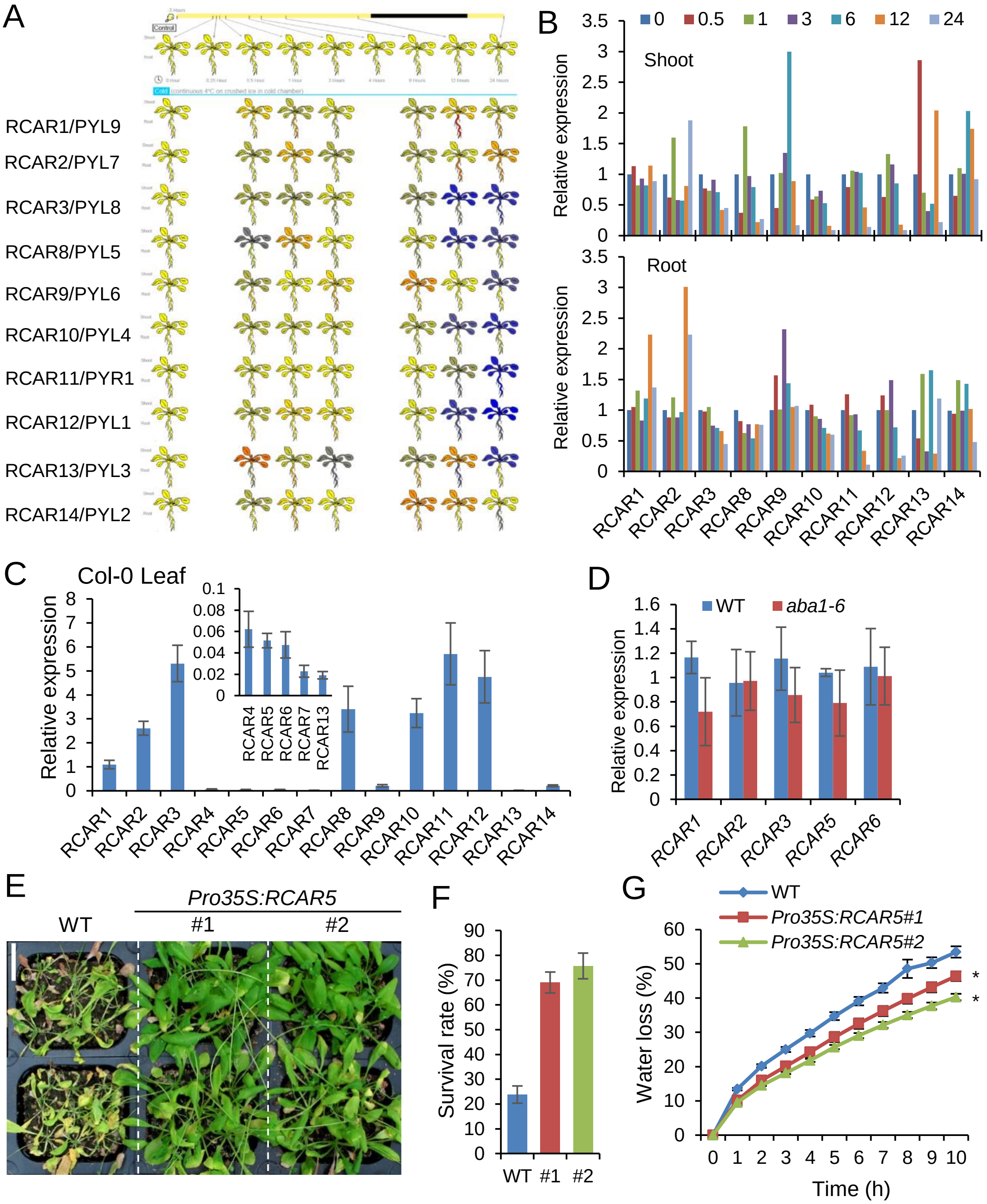
A
RCAR1/PYL9
RCAR2/PYL7
RCAR3/PYL8
RCAR8/PYL5
RCAR9/PYL6
RCAR10/PYL4
RCAR11/PYR1
RCAR12/PYL1
RCAR13/PYL3
RCAR14/PYL2
B
0 0.5 1 3 6 12 24
Shoot
Root
Relative expression
RCAR1 RCAR2 RCAR3 RCAR8 RCAR9 RCAR10 RCAR11 RCAR12 RCAR13 RCAR14
C
Col-0 Leaf
Relative expression
RCAR4 RCAR5 RCAR6 RCAR7 RCAR13
RCAR1 RCAR2 RCAR3 RCAR4 RCAR5 RCAR6 RCAR7 RCAR8 RCAR9 RCAR10 RCAR11 RCAR12 RCAR13 RCAR14
D
WT aba1-6
Relative expression
RCAR1 RCAR2 RCAR3 RCAR5 RCAR6
E
Pro35S:RCAR5
WT #1 #2
F
Survival rate (%)
WT #1 #2
G
WT
Pro35S:RCAR5#1
Pro35S:RCAR5#2
Water loss (%)
Time (h)

Figure S9

FIGURE S9 Expression patterns of *RCAR* genes in Arabidopsis leaves after cold stress treatment. (A) Relative expression levels of *RCAR* genes in the leaves of Arabidopsis ecotype Col-0. *Actin8* was used as an internal control for normalization, and the expression level of *RCAR1* was set to 1.0. (B, C) Relative expression levels of *RCAR* genes in Arabidopsis shoots and roots in response to cold stress. Data were obtained from the Arabidopsis eFP Browser with 'Abiotic stress' (Winter et al., 2007) as the data source in the Bio-Analytic Resource for Plant Biology (http://bar.utoronto.ca/efp/cgi-bin/efpWeb.cgi). (D) Relative expression levels of *RCAR* genes in the leaves of *aba1-6* and WT plants. (E, F) Dehydration sensitivity of *Pro35S:RCAR5* and WT plants. Plants were subjected to dehydration stress by withholding water for 12 days (n = 16); representative images were taken 3 days after rewatering (E) and survival rate was calculated (F). Scale bar= 2 cm. (G) Water loss from leaves of WT and transgenic plants at various times after detachment of leaves. Data represent mean ± standard error of three independent experiments (n = 30). Asterisks indicate significant differences between WT and transgenic plants (Student's *t*-test; $P < 0.05$).
