# Supplementary material for: ABA-Dependent and ABA-Independent Functions of RCAR5/PYL11 in Response to Cold Stress
Source: Front Plant Sci. 2020 Sep 25;11:587620. doi: 10.3389/fpls.2020.587620 (PMC7545830; doi:10.3389/fpls.2020.587620)
Supplement: Supplementary file 11 [file Image_10.pdf]

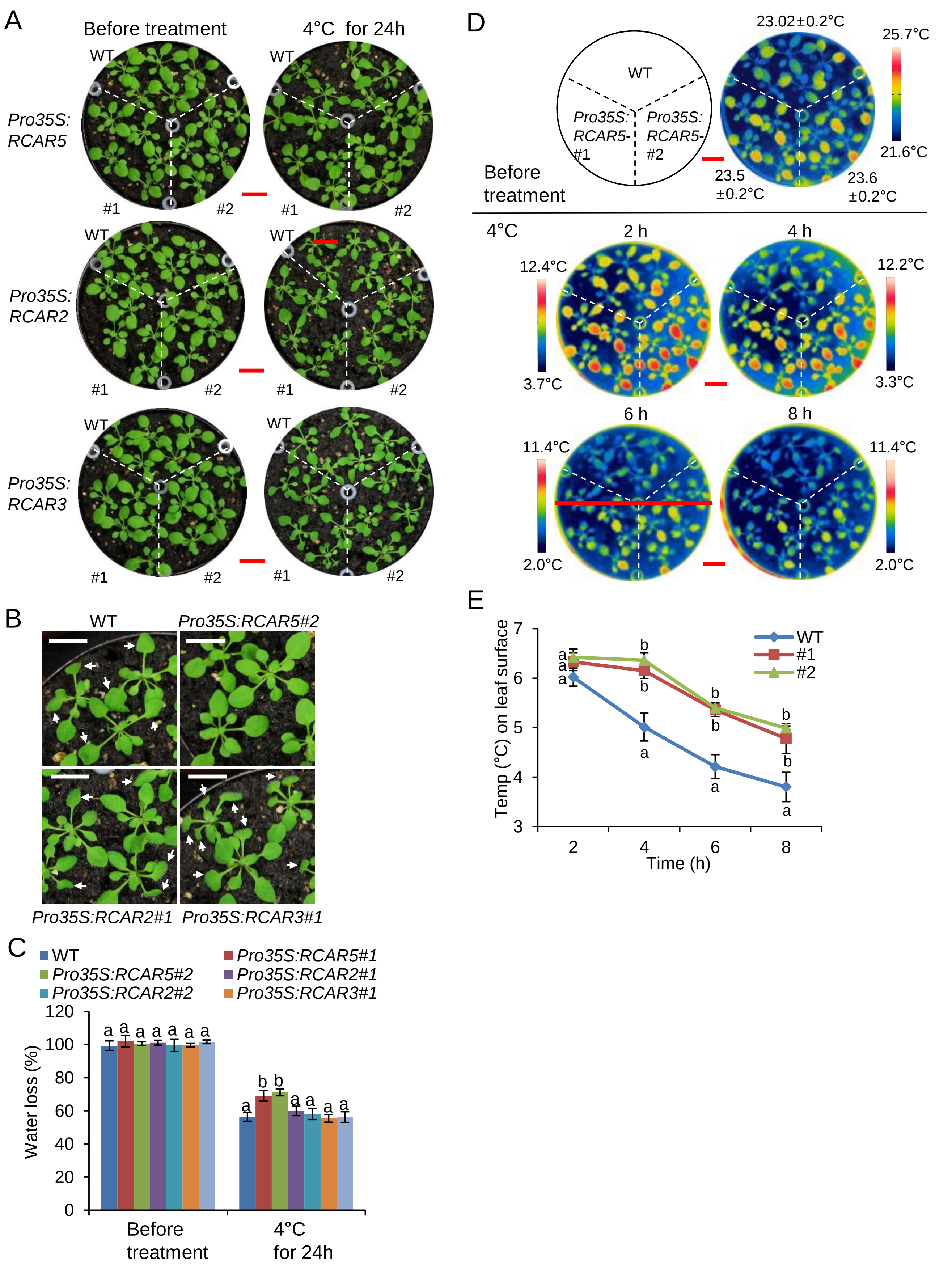
A
Before treatment
4°C for 24h
WT
Pro35S: RCAR5
Pro35S: RCAR2
Pro35S: RCAR3
#1
#2
B
WT
Pro35S:RCAR5#2
Pro35S:RCAR2#1
Pro35S:RCAR3#1
C
WT
Pro35S:RCAR5#1
Pro35S:RCAR5#2
Pro35S:RCAR2#1
Pro35S:RCAR2#2
Pro35S:RCAR3#1
Water loss (%)
0
20
40
60
80
100
120
Before treatment
4°C for 24h
D
WT
Pro35S: RCAR5-#1
Pro35S: RCAR5-#2
Before treatment
23.02±0.2°C
25.7°C
21.6°C
23.5 ±0.2°C
23.6 ±0.2°C
4°C
2 h
4 h
6 h
8 h
12.4°C
3.7°C
12.2°C
3.3°C
11.4°C
2.0°C
E
Temp (°C) on leaf surface
Time (h)
WT
#1
#2
3
4
5
6
7
2
4
6
8

Figure S10

FIGURE S10 Cold stress-induced dehydration of WT and *Pro35S:RCAR2*, *Pro35S:RCAR3*, and *Pro35S:RCAR5* transgenic plants. (A, B) Phenotypic response of WT and transgenic lines to cold stress. Three-week-old Arabidopsis plants (n = 32 per plant line) were exposed to 4°C for 24 h and images were taken (A). Representative parts were magnified (B). Arrow heads indicated wilted leaves. (C) Water loss from WT and *Pro35S:RCAR2*, *Pro35S:RCAR3*, and *Pro35S:RCAR5* plants after cold stress treatment. The fresh weights of each plant line were measured 24 h after treatment. Data represent the mean ± SE of three independent experiments, each evaluating 30 plants. Different letters indicate significant differences between WT and transgenic plants (ANOVA; $P < 0.05$). (D, E) Thermal imaging analysis of *Pro35S:RCAR5* transgenic plants. Representative thermographic images of WT and *Pro35S:RCAR5* plants after cold stress treatment. Thermographic images were taken at different time points after cold stress treatment (D), and the mean leaf temperatures of the two largest leaves were measured using 20 plants of each line (E). Different letters indicate significant differences between WT and transgenic plants (ANOVA; $P < 0.05$). Scale bar= 1 cm.
