# Supplementary material for: ABA-Dependent and ABA-Independent Functions of RCAR5/PYL11 in Response to Cold Stress
Source: Front Plant Sci. 2020 Sep 25;11:587620. doi: 10.3389/fpls.2020.587620 (PMC7545830; doi:10.3389/fpls.2020.587620)
Supplement: Supplementary file 12 [file Image_11.pdf]

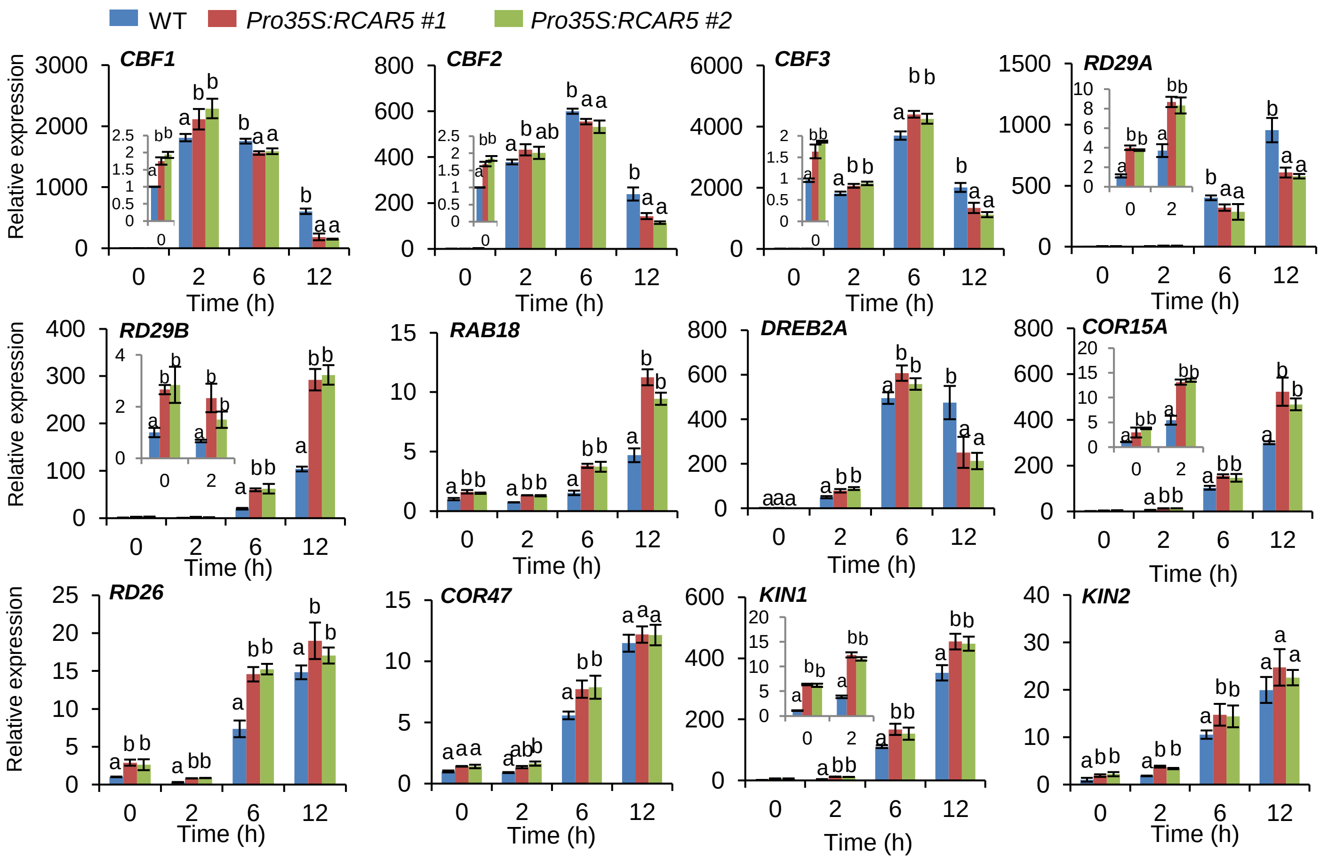


**FIGURE S11** Expression patterns of ABA-responsive genes under cold stress conditions. Two-week-old *Pro35S:RCAR5* and WT plants were exposed to 4°C and shoots were harvested at the indicated time points. *Actin8* were used as an internal control for normalization. The expression level of each gene at 0 h was set to 1.0. Data represent mean ± SD of three independent experiments. Different letters indicate significant difference between WT and transgenic plants (ANOVA; $P < 0.05$).
