# Supplementary material for: ABA-Dependent and ABA-Independent Functions of RCAR5/PYL11 in Response to Cold Stress
Source: Front Plant Sci. 2020 Sep 25;11:587620. doi: 10.3389/fpls.2020.587620 (PMC7545830; doi:10.3389/fpls.2020.587620)
Supplement: Supplementary file 14 [file Image_13.pdf]

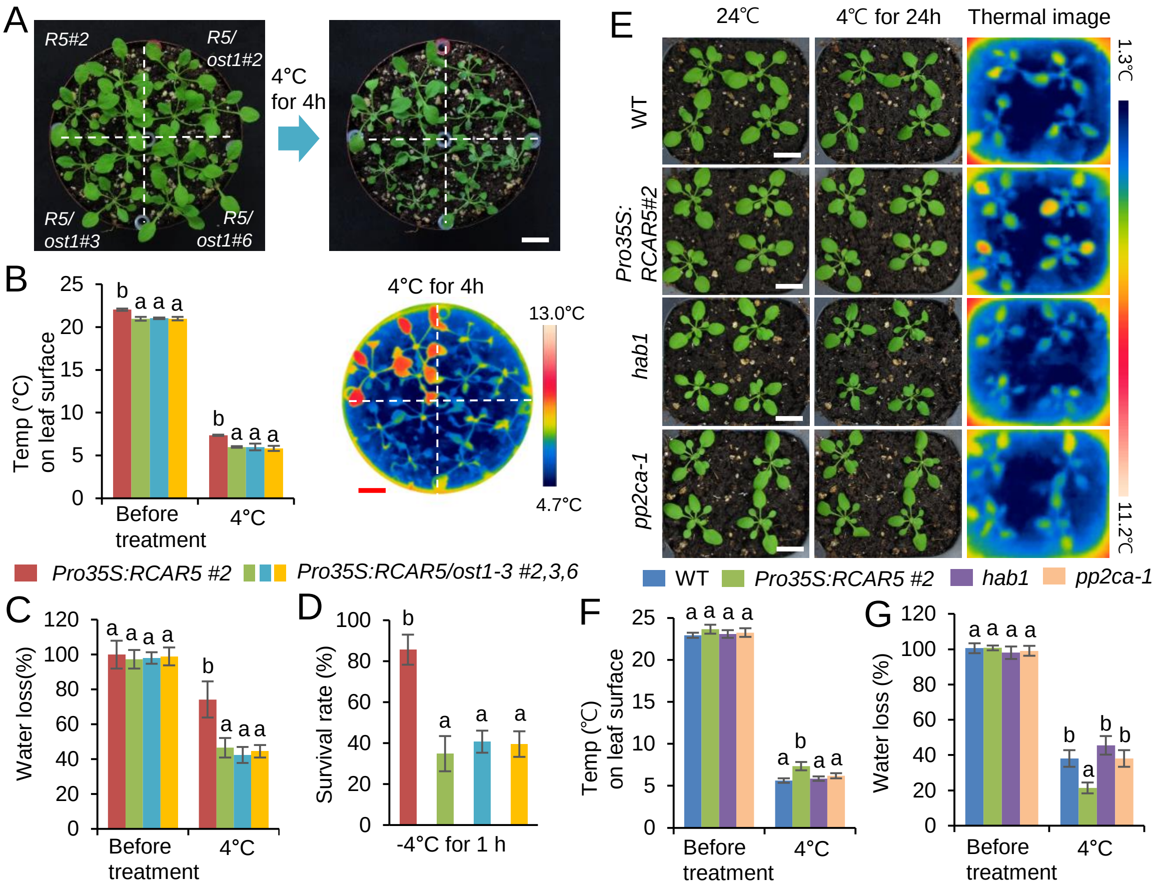

FIGURE S13 Cold stress-induced dehydration of WT and *Pro35S:RCAR5*, *Pro35S:RCAR5/ost1*, *ost1*, *hab1*, and *pp2ca-1* plants. (A, B) Phenotypic response of *Pro35S:RCAR5* and *Pro35S:RCAR5/ost1-3* transgenic lines in response to cold stress. Three-week-old Arabidopsis plants (n=32 per plant line) were exposed to 4°C for 4 h and representative images were taken (A). At the same time, thermographic images were taken (B) and the mean leaf temperatures of the two largest leaves were measured using 20 plants of each line. (C) Water loss from *Pro35S:RCAR5* and *Pro35S:RCAR5/ost1-3* plants after cold stress treatment. The fresh weights of each plant line (n = 30) were measured 24 h after treatment. (D) Freezing tolerance of *Pro35S:RCAR5* and *Pro35S:RCAR5/ost1-3* plants. Three-week-old seedlings of each plant line were exposed to -4°C for 1 h. After recovery at 24°C for 2 days, the survival rate of each line was counted. (E-G) Leaf dehydration and leaf temperatures of *Pro35S:RCAR5*, *hab1*, *pp2ca-1*, and WT plants in response to cold stress. Representative thermographic images of *Pro35S:RCAR5*, *hab1*, *pp2ca-1*, and WT plants 4 h after exposure to 4°C (E); the mean leaf temperatures of the two largest leaves were measured using 20 plants of each line (F). Concomitantly, the fresh weights of each plant line were measured (G). All data represent mean ± SD of three independent experiments. Different letters indicate significant differences between WT and transgenic plants (ANOVA; $P < 0.05$). Scale bar= 1 cm.
